# The tumor suppressor miR-642a-5p targets Wilms Tumor 1 gene and cell-cycle progression in prostate cancer

**DOI:** 10.1038/s41598-021-97190-x

**Published:** 2021-09-09

**Authors:** Dianne J. Beveridge, Kirsty L. Richardson, Michael R. Epis, Rikki A. M. Brown, Lisa M. Stuart, Andrew J. Woo, Peter J. Leedman

**Affiliations:** 1 Laboratory for Cancer Medicine, Harry Perkins Institute of Medical Research, QEII Medical Centre, 6 Verdun St, Nedlands, 6009 Australia; 2 Centre for Medical Research, The University of Western Australia, Crawley, WA 6009 Australia; 3 School of Medical and Health Sciences, Edith Cowan University, Joondalup, WA 6027 Australia; 4 School of Medicine and Pharmacology, The University of Western Australia, Crawley, WA 6009 Australia

**Keywords:** Prostate cancer, Drug development

## Abstract

RNA-based therapeutics are emerging as innovative options for cancer treatment, with microRNAs being attractive targets for therapy development. We previously implicated microRNA-642a-5p (miR-642a-5p) as a tumor suppressor in prostate cancer (PCa), and here we characterize its mode of action, using 22Rv1 PCa cells. In an in vivo xenograft tumor model, miR-642a-5p induced a significant decrease in tumor growth, compared to negative control. Using RNA-Sequencing, we identified gene targets of miR-642a-5p which were enriched for gene sets controlling cell cycle; downregulated genes included Wilms Tumor 1 gene (WT1), NUAK1, RASSF3 and SKP2; and upregulated genes included IGFBP3 and GPS2. Analysis of PCa patient datasets showed a higher expression of WT1, NUAK1, RASSF3 and SKP2; and a lower expression of GPS2 and IGFBP3 in PCa tissue compared to non-malignant prostate tissue. We confirmed the prostatic oncogene WT1, as a direct target of miR-642a-5p, and treatment of 22Rv1 and LNCaP PCa cells with WT1 siRNA or a small molecule inhibitor of WT1 reduced cell proliferation. Taken together, these data provide insight into the molecular mechanisms by which miR-642a-5p acts as a tumor suppressor in PCa, an effect partially mediated by regulating genes involved in cell cycle control; and restoration of miR-642-5p in PCa could represent a novel therapeutic approach.

## Introduction

Prostate cancer (PCa) is the second most diagnosed cancer worldwide accounting for 3.8% of cancer related death in men^1^. PCa diagnoses have increased in recent years, which is attributable to both the broader awareness of the disease, improved detection methods, and the emergence of screening for biomarkers (e.g. Prostate-Specific Antigen (PSA))^2^. There has also been a significant increase in the early diagnosis of localized, low-risk PCa, ranging from 10 to 80% of all men diagnosed with PCa worldwide^3^, with a subsequent decrease in PCa mortality^4^. A sizeable proportion of men with low-risk PCa are carefully monitored via active surveillance and do not require treatment or surgery^5,6^. PCa growth is initially androgen-dependant via the expression of the androgen receptor (AR), providing the basis for androgen deprivation therapies. In the last decade, the development of multiple drugs that target the androgen axis has improved the survival of PCa patients, including Abiraterone Acetate and Enzalutamide^7–9^. Unfortunately in many men, the disease transforms into hormone refractory or castrate resistant prostate cancer (CRPC), whereby tumors become increasingly resistant to conventional AR pathway inhibitor treatments characterized by metastasis and premature death^10,11^. Thus, there remains a large unmet clinical need to develop novel approaches to treat PCa, especially advanced CRPC.

MicroRNAs (miRNAs) are a family of ~ 22 nucleotide noncoding RNAs that are powerful regulators of gene expression via targeting of the 3′untranslated region (3′UTR) of specific genes leading to translational repression or message decay^12^. With their aberrant expression known to play a pivotal role in the regulation of a variety of developmental processes and diseases, miRNAs have therapeutic potential for the treatment of cancer and other illnesses^13^. Several miRNA-targeted therapies have reached clinical development, including miR-34 (in the form of a double-stranded miRNA mimic) for treating cancer, and miR-122 (in the form of antimiRs) for treating hepatitis C^14,15^. There is aberrant miRNA expression in cancer, leading to both inhibition and promotion of the tumorigenic process, with respective loss of expression of tumor suppressor miRNAs or overexpression of oncogenic miRNAs (oncomiRs)^16–18^. In PCa, there has been the identification of miRNA signatures associated with either poor prognosis or response to therapy, and some have potential functional roles as biomarkers^19–24^. These studies emphasize the potential for miRNAs to become cancer therapeutics, and provides an opportunity to identify downregulated tumor suppressor miRNAs, the replacement of which could be a new strategy in the treatment of PCa.

Previously, we discovered that miR-642a-5p is a tumor suppressor in PCa^25^. We showed overexpression of miR-642a-5p in PCa cells resulted in reduced cell viability, and deoxyhypusine hydroxylase (DOHH) to be a direct target of miR-642a-5p. DOHH catalyzes the activation of eukaryotic translation initiation factor (eIF5A), a protein essential for cell growth, and therefore the targeting of DOHH by miR-642a-5p resulted in less eIF5A activity and a reduction in cell proliferation. Additionally, we found miR-642a-5p to be downregulated in PCa cell lines or tissue, relative to matched normal cells or tissue, the expression of which was not attributable to the hyper methylation of its promoter^25^.

Here, we investigated the mode of action of miR-642a-5p in PCa, and aimed to identify novel downstream targets of miR-642a-5p, to further understand its effect as a tumor suppressor and potential as a prospective PCa therapeutic. In an in vivo xenograft model of PCa, transient overexpression of miR-642a-5p potently reduced tumor growth. RNA-Sequencing (RNA-Seq) analysis of miR-642a-5p treated PCa cells identified dysregulation of genes that cluster to specific pathways including cancer, cell cycle, organismal injury and abnormalities, and cellular growth and proliferation. Further, we identified for the first time Wilms Tumor 1 gene (WT1), which is an oncogene in PCa^26,27^, as a new direct target of miR-642a-5p in PCa, providing novel insight into the role of miR-642a-5p as a tumor suppressor in PCa. Taken together, in PCa, miR-642a-5p has broad anti-tumor activity acting on several tumor pathways, and specifically on genes that regulate proliferation and cell cycle progression.

## Results

### miR-642a-5p inhibits prostate cancer xenograft tumor growth and increases survival

**Figure 1:**
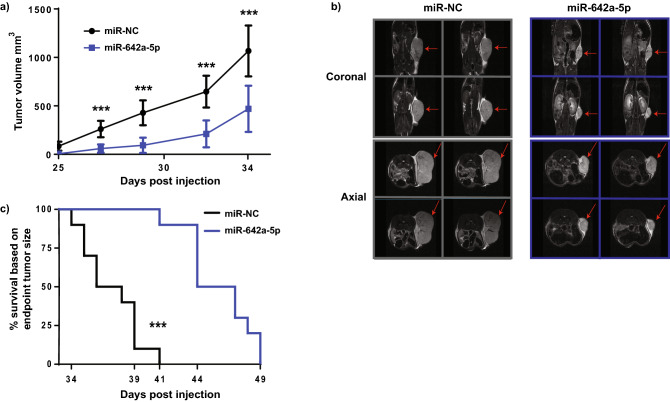
miR-642a-5p inhibits PCa xenograft tumor growth and increases survival. Subcutaneous xenograft study of 22Rv1 PCa cells transiently overexpressing miR-642a-5p or miR-NC in NSG mice (10 per group). (**a**) Xenograft tumor volumes in mice measurable from day 25 to day 34. CI = 0.95; ***p < 0.0002. (**b**) Coronal and axial MRI images of day 34 representative mice from both miR-NC and miR-642a-5p xenograft groups. Red arrows indicate tumor. (**c**) Tumor size end point Kaplan–Meier survival curve of miR-642a-5p versus miR-NC xenograft mice. Log-rank (Mantel-Cox) Test ***p < 0.0001, Gehan–Breslow–Wilcoxon Test ***p < 0.0002.

In order to investigate the effect of miR-642a-5p on PCa cell growth in vivo, we transiently overexpressed miR-642a-5p or a negative control miRNA (miR-NC) in human 22Rv1 PCa cells (representative of castrate resistant disease^28^), and subcutaneously transplanted them into male NOD/SCID gamma (NSG) mice to generate xenografts (10 mice/group). Subsequent to day 25-post injection, we observed a rapid increase in xenograft tumor volume in the miR-NC mice compared to the miR-642a-5p mice (Fig. 1a). Coronal and axial magnetic resonance imaging (MRI) of animals at 34 days post injection also corroborated the differences in tumor volume observed between the two groups (Fig. 1b). The end point based on tumor size (1500 mm^3^) was reached first by mice in the miR-NC transfected group at day 34 post injection, and all miR-NC xenografts reached end point by day 41 (Fig. 1c). In contrast, only one of the 10 mice in the miR-642a-5p group reached end point at day 41, with the remainder of the mice in this group progressively reaching end point by day 49 (Fig. 1c). The tumor size-based survival of the miR-642a-5p treated mice was significantly different to the miR-NC treated mice, as determined by Log-rank (Mantel-Cox) and Gehan–Breslow–Wilcoxon analyses (*p* < 0.0001; *p* < 0.0002, respectively). Taken together, these data indicate that transient overexpression of miR-642a-5p significantly inhibits PCa xenograft growth and is associated with increased survival.

### RNA-Sequencing target gene identification and pathway analysis

**Figure 2:**
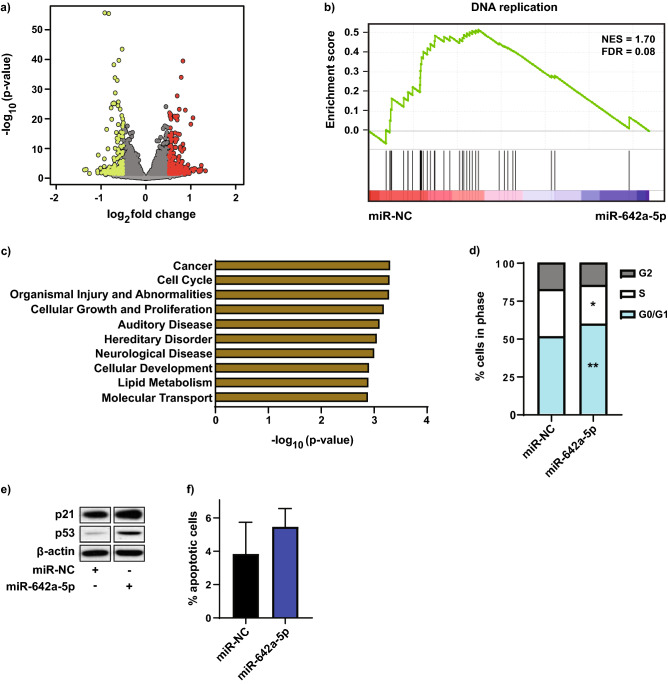
miR-642a-5p targets genes and pathways involved in DNA replication and cell cycle arrest. (**a**) Volcano plot of RNA-Seq results, with green dots representing genes downregulated, and red dots representing genes upregulated (≥ 0.5 log_2_ fold change; *p* < 0.05) by miR-642a-5p. (**b**) Gene Set Enrichment Analysis (GSEA) of the RNA-Seq data. The y-axis and the green line show the enrichment score for each gene, illustrated as a vertical line plotted in rank order of the most gene abundance (red, left) to the least gene abundance (blue, right) within the indicated samples (as log_2_FC/comparison); the black vertical lines correspond to member genes from the set. NES normalized enrichment score, FDR false discovery rate. (**c**) Ingenuity Pathway Analysis of the miR-642a-5p up and downregulated genes. Pathways ranked by the *p* value result of a Fisher’s exact test. (**d**) Flow cytometry cell cycle analysis of 22Rv1 cells transfected with miR-642a-5p or miR-NC (30 nM) for 72 h. n = 3; *p < 0.05, **p < 0.005 relative to miR-NC. (**e**) Western blot analysis of p21 and p53 protein expression 72 h post-transfection of 22Rv1 cells with 30 nM miR-642a-5p or miR-NC. β-actin is the loading control. Bands are from non-adjacent lanes of the same western blot and are separated by white space (see Supplementary Fig. S1A for uncropped blots, which were cut into smaller strips prior to immunoblotting). n = 3. (**f**) Flow cytometry apoptosis analysis of 22Rv1 cells transfected miR-642a-5p or miR-NC (30 nM) for 72 h. Error bars = SD; n = 3; *p* > 0.05.

To explore the mechanism of miR-642a-5p’s potent inhibition of 22Rv1 PCa xenograft growth, we performed RNA-Seq analysis of 22Rv1 cells treated with miR-642a-5p or miR-NC (30 nM for 24 h). Using ≥ 0.5 log_2_ fold change and *p* < 0.05, we identified 448 genes that were differentially expressed between the two groups; 176 genes were downregulated and 272 were upregulated (Fig. 2a and Supplementary Tables S1 and S2). Of the 176 genes that were downregulated by miR-642a-5p overexpression, 72 (~ 41%) contained at least one miR-642a-5p seed site (via TargetScan 7.2), and these genes are listed in Supplementary Table S3. DOHH, which we previously identified as a direct target of miR-642a-5p, was ranked at the top of Supplementary Table S3 having 6 seed sites and was downregulated 0.69 log_2_ fold (^25^ and Supplementary Table S3). Interestingly, of the 272 genes that were upregulated by miR-642a-5p overexpression, 47 (~ 17%) also contained at least one miR-642a-5p seed site, and these genes are listed in Supplementary Table S4 and are putative indirect targets of miR-642a-5p.

To ascertain the major gene networks and biological pathways regulated by miR-642a-5p in 22Rv1 cells, we performed Gene Set Enrichment Analysis (GSEA) and Ingenuity Pathway Analysis (IPA) of the 448 differentially expressed genes from the RNA-Seq data. The GSEA analysis identified the KEGG (Kyoto Encyclopedia of Genes and Genomes)^29^ pathway ‘DNA replication’ as a significantly depleted gene network following miR-642a-5p overexpression (Fig. 2b). We identified cancer, cell cycle, organismal injury and abnormalities, and cellular growth and proliferation as the most significantly enriched biological processes (ranked by the p-value result of a Fisher’s exact test) with miR-642a-5p treatment (Fig. 2c).

### Effect of miR-642a-5p on cell cycle progression

To further investigate the effect of miR-642a-5p on the cell cycle (Fig. 2c), we performed flow cytometry cell cycle analysis of 22Rv1 cells transfected with miR-642a-5p or miR-NC, and found that miR-642a-5p induces cell cycle arrest at G0/G1 and a block in transition to S phase (Fig. 2d). We next overexpressed miR-642a-5p in 22Rv1 cells, and consistent with these cells harboring wild-type p53^30^, we observed an increase in the expression of the tumor suppressor cell cycle inhibitors p53 and p21, which supports the observed cell cycle arrest at G0/G1 (Fig. 2e). We then determined if miR-642a-5p overexpression affects apoptosis, and following annexin V, propidium iodide (PI) staining and flow cytometry, there was an increase in the apoptotic fraction of miR-642a-5p treated cells, however this difference was not statistically significant (*p* > 0.05) (Fig. 2f). Taken together, these data support the notion that mediation of the growth inhibitory action of miR-642a-5p in 22Rv1 PCa cells is, in part, via alteration of cell cycle progression.

### Identification of genes regulated by miR-642a-5p and implicated in cell cycle arrest

**Figure 3:**
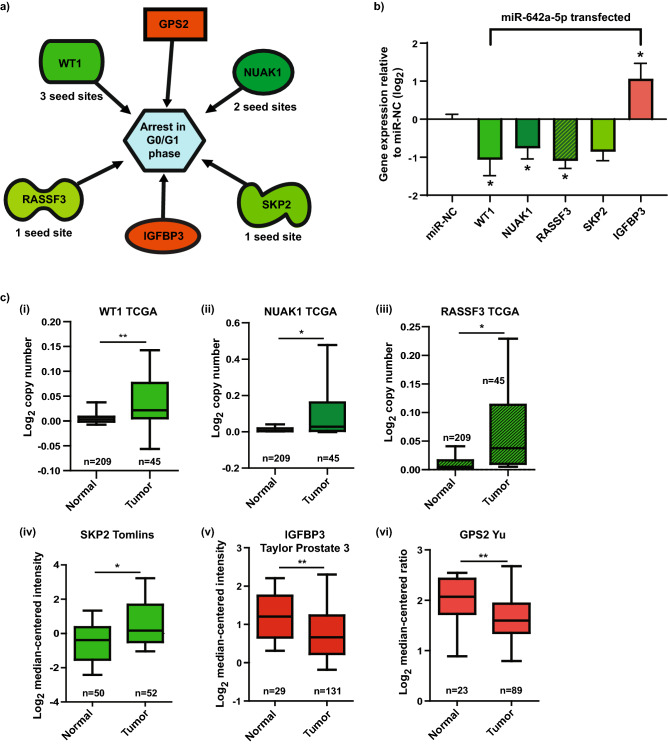
Cell cycle arrest gene targets of miR-642a-5p. (**a**) Ingenuity Pathway Analysis of cell cycle targets of miR-642a-5p. Green denotes genes downregulated by miR-642a-5p, and the number of seed sites in their 3’UTR (identified by TargetScan 7.2) indicated. Red denotes genes upregulated by miR-642a-5p. (**b**) RT-qPCR analysis of the cell cycle genes following overexpression of miR-642a-5p in 22Rv1 PCa cells. Expression of target mRNAs is normalized to HPRT housekeeping gene expression, calculated using the 2^−ΔΔCt^ method, and relative to miR-NC. Error bars = SE; n = 3; *p < 0.05 relative to miR-NC. (**c**) Oncomine analysis of the expression of the miR-642a-5p targets in PCa data sets. (i) WT1; (ii) NUAK1; (iii) RASSF3; (iv) SKP2; (v) IGFBP3; and (vi) GPS2. The data cohorts indicated above each graph, and n per group shown. Boxes denote the median (horizontal line); whiskers indicate distances to the highest and lowest values [for NUAK1 and RASSF3 the lower whisker is to the 10th percentile (minimum value removed)]. *p < 0.05, **p < 0.005.

Further interrogation of the RNA-Seq data by IPA revealed a number of novel putative cell cycle associated gene targets for miR-642a-5p, which were either significantly (*p* < 0.05) downregulated or upregulated ≥ 0.5 log_2_ fold. Downregulated genes included WT1, NUAK1 [NUAK family SNF1-like kinase 1; also known as AMPK-related protein kinase 5 (ARK5)], RASSF3 (Ras association domain family member 3), and SKP2 (S-phase kinase-associated protein 2), and are indicated in green in Fig. 3a. Conversely, GPS2 (G Protein Pathway Suppressor 2; also known as AMF1) and IGFBP3 (Insulin-like growth factor-binding protein 3) indicated in red in Fig. 3a were upregulated by miR-642a-5p in the RNA-Seq and are all associated with G0/G1 arrest (IPA). We further validated these findings by transiently overexpressing miR-642a-5p or miR-NC in 22Rv1 cells and measured the mRNA expression of these cell cycle genes. The expression of WT1, NUAK1, RASSF3, and SKP2 were all downregulated, and the expression of IGFBP3 was upregulated following miR-642a-5p treatment (Fig. 3b). There was no significant difference in GPS2 mRNA levels following miR-642a-5p overexpression (data not shown). Taken together, these data support the regulation of cell cycle genes as a key proposed mechanism of miR-642a-5p action in PCa cells.

### Clinical impact of the miR-642a-5p cell cycle targets

To explore the potential clinical impact of these data we used Oncomine analysis to interrogate various PCa datasets and compared the gene expression of our miR-642a-5p targets between normal prostate and PCa samples. Examination of these cohorts revealed that each of the downregulated miR-642a-5p target genes (WT1, NUAK1, RASSF3, and SKP2) is a potential driver of tumor progression, as each of their expression levels was higher in prostate tumor samples when compared to normal prostate tissue (Fig. 3c i–iv). Conversely, both IGFBP3 and GPS2, which were upregulated with miR-642a-5p overexpression in RNA-Seq, had lower levels of expression in the PCa samples (Fig. 3c v and vi). This data suggests that miR-642a-5p coordinately regulates (either up or down) a range of genes, with the net result of substantially decreasing PCa cell growth.

### Validation of WT1 as a direct target of miR-642a-5p

**Figure 4:**
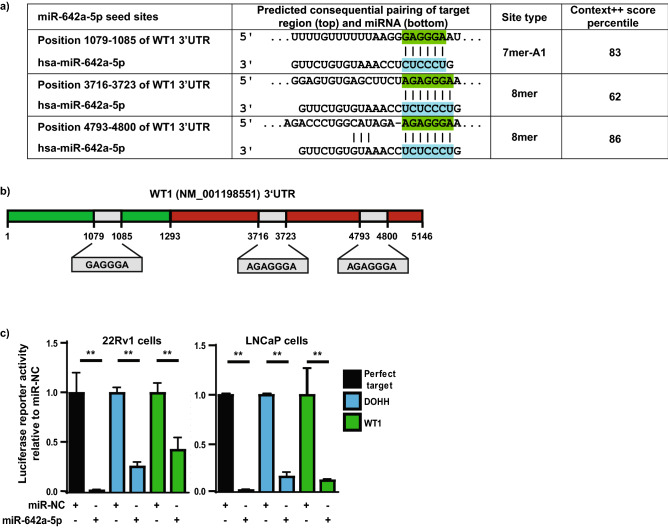
WT1 is a direct target of miR-642a-5p in PCa cells. (**a**) The 3′UTR of WT1 has three putative miR-642a-5p seed sites as predicted by TargetScan 7.2. (**b**) Schematic of the 3′UTR of WT1 (not to scale). Depiction of the GeneCopoeia target clone, which contains only the first 1293 base pairs of the 3′UTR, is with green shading. The grey shaded boxes indicate the miR-642a-5p seed sites. (**c**) Luciferase reporter gene analysis of the 3′UTR of the putative miR-642a-5p target WT1 in 22Rv1 and LNCaP PCa cells transiently overexpressing miR-642a-5p or miR-NC (20 nM). DOHH and miR-642a-5p perfect targets are positive controls. Error bars = SD; n = 3; **p < 0.005.

There is increasing evidence suggesting that WT1 functions as an oncogene in PCa^26^, and given its involvement in cell cycle progression^27^, the existence of three miR-642a-5p seed sites within its 3′UTR (Fig. 4a and Supplementary Table S3), and its clinical impact [Fig. 3c(i)], we chose to evaluate WT1 further as a target of miR-642a-5p. To ascertain whether WT1 is a direct target of miR-642a-5p, we transiently co-transfected a luciferase reporter construct containing the first 1293 base pairs (bp) of WT1’s 3′UTR region (total length 5146 bp) (Fig. 4b), along with miR-642a-5p or miR-NC into 22Rv1 and LNCaP PCa cells. Transfection of miR-642a-5p significantly (*p* < 0.005) downregulated the luciferase reporter activity of WT1 3′UTR in both of the PCa cell lines validating the direct targeting of miR-642a-5p (Fig. 4c). As positive controls, we included the DOHH 3′UTR construct, as well as a reporter containing the perfect target sequence for miR-642a-5p^25^. Together with the TargetScan prediction of three seed sites, this data is the first evidence supporting the concept that WT1 is a direct target of miR-642a-5p, and therefore an important downstream target of miR-642a-5p.

### Targeted siRNA knockdown of WT1 reduces cell proliferation and blocks cell cycle progression

**Figure 5:**
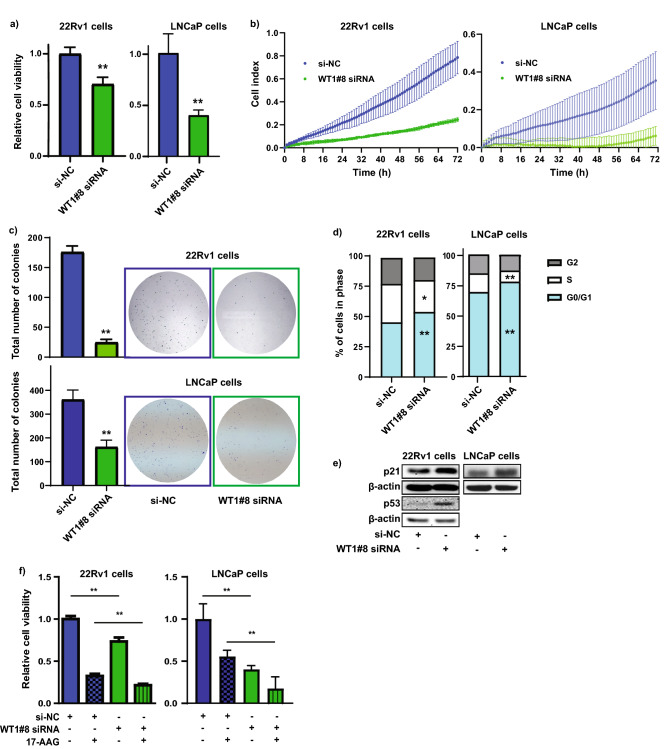
Targeted siRNA-mediated inhibition of WT1 expression reduces PCa cell proliferation and blocks cell cycle progression. (**a**) Relative cell viability of 22Rv and LNCaP PCa cells measured via cell titer assay at 3 d post-transfection with WT1 siRNA or si-NC (20 nM). Validation of WT1 knockdown see Fig. S2B. Error bars = SD; n = 3; **p < 0.005. (**b**) Proliferation of 22Rv1 and LNCaP PCa cells (cell index) measured using the xCELLigence system post WT1 siRNA or si-NC transfection (20 nM). Validation of WT1 knockdown see Fig. S2C. Error bars = SD; n = 3. (**c**) Colony formation assay of 22Rv1 and LNCaP PCa cells 14–21 days post WT1 siRNA or si-NC (20 nM) transfection. Validation of WT1 knockdown see Fig. S2C. Error bars = SD; n = 3; **p < 0.005. (**d**) Flow cytometry cell cycle analysis of 22Rv1 and LNCaP PCa cells transfected with WT1 siRNA or si-NC (20 nM) for 72 h. Validation of WT1 knockdown see Fig. S1D. n = 3; *p < 0.05, **p < 0.005 relative to si-NC. (**e**) Western blot analysis of p21 (22Rv1 and LNCaP) and p53 (22Rv1) protein expression 72 h post-transfection of PCa cells with 20 nM WT1 siRNA or si-NC. β-actin is the loading control. Validation of WT1 knockdown see Fig. S2E. For full-length, non-cropped blots see Fig. S1B and S1C. n = 3. (**f**) Relative cell viability of 22Rv1 and LNCaP PCa cells measured via cell titer assay at 5 days post-transfection with WT1 siRNA or si-NC (20 nM), and 3 d post-17-AAG treatment (1 µM). Validation of WT1 knockdown see Fig. S2B. Error bars = SD; n = 3; **p < 0.005.

We next used small interfering RNA (siRNA) to transiently knockdown WT1 gene expression in 22Rv1 and LNCaP PCa cells, to assess the functional effects of WT1 on PCa growth. We initially tested four different WT1 siRNAs in 22Rv1 cells, and RT-qPCR quantitation confirmed an approximate 80% reduction in WT1 expression with the ‘WT1#8’ siRNA, as compared to negative control siRNA (si-NC) (Supplementary Fig. S2A). We subsequently used WT1#8 siRNA in our experiments, and in each instance validated WT1 knockdown via RT-qPCR (Supplementary Fig. S2B–E). We transiently transfected 22Rv1 and LNCaP cells with either WT1 siRNA or si-NC and assessed cell proliferation using a Cell Titer end-point assay or the xCELLigence real time system. WT1 siRNA transfected cells exhibited a substantial growth reduction as compared to the si-NC transfected cells using both methods of evaluation (Fig. 5a, b). Furthermore, siRNA-mediated knockdown of WT1 reduced colony formation in clonogenicity assays (Fig. 5c). We also performed flow cytometry cell cycle analysis of 22Rv1 and LNCaP PCa cells transfected with WT1 siRNA or si-NC, and found that WT1 knockdown induced cell cycle arrest at G0/G1, and a concurrent increase in p21 and p53 expression (Fig. 5d, e). Additionally, treatment of 22Rv1 and LNCaP PCa cells with a combination of both WT1 siRNA and a clinically available inhibitor of WT1 (Tanespimycin (17-AAG [17-(allylamino)-17-demethoxygeldanamycin]), resulted in a further reduction of cell growth than with WT1 siRNA or 17-AAG alone (Fig. 5f). Taken together, these data suggest that therapeutic targeting of WT1 in PCa could be beneficial for tumor growth inhibition.

### Overexpression of WT1 increases colony formation and miR-642a-5p rescues this effect

**Figure 6:**
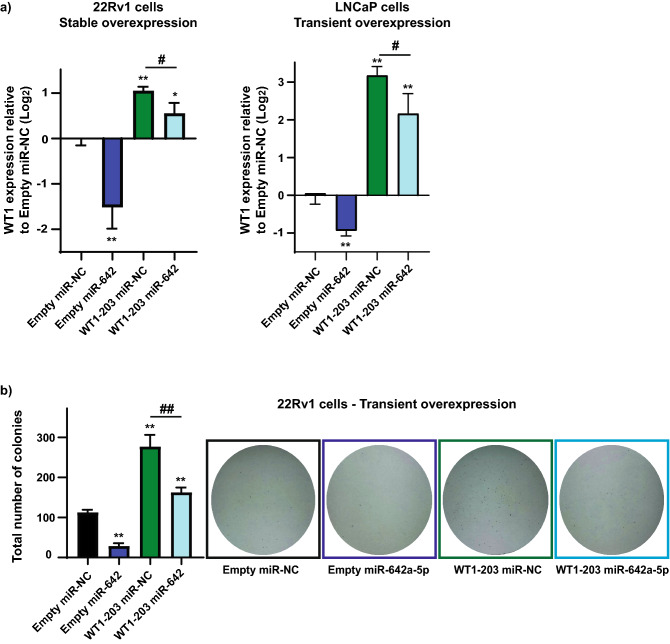
WT1 overexpression increases colony formation and miR-642a-5p rescues this effect. (**a**) RT-qPCR analysis of WT1 gene expression following stable or transient LeGO-iT2-WT1-203 or LeGO-iT2-Empty plasmids, and overexpression of miR-642a-5p or miR-NC in 22Rv1 and LNCaP PCa cells. Expression of WT1 is normalized to HPRT housekeeping gene expression, calculated using the 2^−ΔΔCt^ method, and relative to Empty vector + miR-NC. Error bars = SE; n = 3; *p < 0.05, **p < 0.005 relative to Empty vector + miR-NC. ^#^*p* < 0.05 WT1-203 + miR-NC relative to WT1-203 + miR-642a-5p. (**b**) Colony formation assay of 22Rv1 PCa cells 14 days post transient WT1 overexpression/empty vector transfection and miR-NC/miR-642a-5p (30 nM) co-transfection. Error bars = SD; n = 3; **p < 0.005 relative to Empty vector + miR-NC. ^##^*p* < 0.005 WT1-203 + miR-NC relative to WT1-203 + miR-642a-5p.

To further investigate the anti-cancer contribution of WT1 targeting by miR-642a-5p, we transiently and stably overexpressed WT1 cDNA (WT1-203 isoform) in 22Rv1 and LNCaP PCa cells, and transfected these cells with 30 nM miR-642a-5p or miR-NC. RT-qPCR analysis validated the stable or transient WT1 overexpression, and subsequent miR-642a-5p treatment significantly downregulated WT1 expression (Fig. 6a). Overexpression of WT1 in 22Rv1 PCa cells resulted in an increase in colony formation in clonogenicity assays, an effect which was rescued with miR-642a-5p overexpression (Fig. 6b). Taken together, miR-642a-5p replacement in 22Rv1 and LNCaP PCa cells with ectopic overexpression of WT1 significantly ‘rescues’ its anti-cancer effects on WT1 gene targeting, further suggesting miR-642a-5p could be an ideal therapy in PCa.

## Discussion

There have been numerous reports regarding the molecular signatures and functions of specific microRNAs in cancer, and there are important opportunities to identify tumor suppressor microRNAs^15,31–33^. There is little known about the functional role of miR-642a-5p and cancer, and here we characterize its mode of action as a tumor suppressor in 22Rv1 and LNCaP PCa cells, both being models of CRPC^34^. Overexpression of miR-642a-5p resulted in a considerable decrease in xenograft tumor growth in vivo, and its overexpression dysregulated genes involved in DNA replication and cell cycle progression. Further, the expression of the cell-cycle-regulated genes which were either downregulated (e.g. WT1, NUAK1, RASSF3, and SKP2) or upregulated (e.g. GPS2 and IGFBP3) by miR-642a-5p in PCa were inversely related to the effect of the miRNA; those genes downregulated by miR-642a-5p were upregulated in tumor compared to normal prostate and vice versa, further supporting miR-642a-5p as a tumor suppressor microRNA. Additionally, our data suggest that direct therapeutic targeting of the miR-642a-5p cell cycle target genes, in particular WT1, could produce significant anti-tumor effects to benefit PCa patients.

We first described miR-642a-5p as a tumor suppressor in PCa^25^, and indeed, there are few studies exploring the role of miR-642a-5p in cancer. In one report, expression of miR-642a-5p was downregulated in colon cancer cell lines and tumor tissue compared to normal, and overexpression of miR-642a-5p reduced the growth of colon cancer cell lines in vitro and in vivo^35^, further supporting our observations of miR-642a-5p as a tumor suppressor. Interestingly, also in colon cancer, the long non-coding RNA LINC01234 and the circular RNA-103809 have been shown to act as competing endogenous RNAs (ceRNA) or ‘sponges’ of miR-642a-5p, thereby reducing its bioavailability and tumor suppressive functions^35,36^. Additionally, the expression of miR-642a-5p was reduced in colorectal cancer cells, in peripheral immune cells following resection of lung tumors, and in childhood hematological cancers suggesting a potential biomarker role of miR-642a-5p in the diagnosis of these cancers^37–39^. Another recent report demonstrated a tumor suppressive function of miR-642a in liver cancer (hepatocellular carcinoma (HCC)); miR-642a expression was decreased, which enabled increased SEMA4C expression and signaling via the p38 MAPK pathway^40^. Also in HCC, Tang and colleagues showed miR-642 to be a tumor suppressor ceRNA, via interacting with, and disrupting the oncogenic functions of Linc00974 and KRT19^41^. In a study investigating advanced bladder cancer, expression of miR-642a-5p was reduced in patient tumors, and transient overexpression of miR-642a-5p mimics in bladder cancer cells in vitro reduced their viability, consistent with a tumor suppressor role^42^. Taken together, these studies provide increasing evidence that miR-642a-5p is a potent tumor suppressor across several cancer types.

Dysregulation of cell cycle and its control underpins cancer biogenesis and its capacity to proliferate^43^. Our data demonstrate that miR-642a-5p overexpression regulates a coordinated set of genes that drive cell cycle arrest at the G0/G1 (proliferation or quiescence) phase, and support cell cycle arrest as a proposed key mechanism of miR-642a-5p action. Cyclin-dependent kinases (CDKs) are critical enzymes that promote transition through the cell cycle and hence the targeting of these in proliferating cancer cells has been the basis for development and clinical application of novel anticancer therapies^43–45^. The CDK inhibitors p21 and p53 are known tumor suppressors and play key roles in regulating transition of cells through the cell cycle^46^. Our data showing their upregulation following miR-642a-5p overexpression also support the role of miR-642a-5p as a tumor suppressor in PCa. Interestingly, miR-642a-5p’s ability to reduce cell viability of PCa cells is not reliant on cellular p53 status, as our previous study using other PCa cell subtypes which harbour non-functional p53 (e.g. DU145) also showed a reduction in cell viability with miR-642a-5p overexpression^25,30^.

Wilms Tumor 1 (WT1) gene is a member of the early growth response gene I (EGR-1) family of zinc finger transcription factors, having an important role in the normal development of the genitourinary system and other organs and tissues^47,48^. In addition to the requisite role of WT1 in development, it also plays a complex role in tumorigenesis, acting as either a tumor suppressor or an oncogene depending on the cellular context^47,48^. There is mounting evidence that WT1 functions as an oncogene in PCa, acting by facilitating the development of a lethal metastatic phenotype^26,27^. In addition, WT1 expression is elevated in high-grade PCa tissues, and the level of expression may serve as a biomarker for PCa progression^49^. Furthermore, administration of 17-AAG, a clinically available inhibitor of WT1 (via its interaction with heat shock protein 90), was shown to decrease myeloid leukemia xenograft growth, correlating with decreased expression of WT1 and its downstream targets^50^. Our data suggests WT1 is a direct target of miR-642a-5p, and siRNA or 17-AAG targeting of WT1 reduced cellular proliferation in 22Rv1 and LNCaP PCa cells. Furthermore, when WT1 was overexpressed in 22Rv1 and LNCaP cells miR-642a-5p overexpression effectively reduced WT1 gene expression and colony formation. These data, together with our TCGA Oncomine data, support the concept that therapies targeting WT1, such as miR-642a-5p replacement treatment or 17-AAG, could reduce PCa growth and potentially represent treatment alternatives.

The other putative miR-642a-5p target genes, which are associated with cell cycle, have roles as oncogenes or tumor suppressors in cancer. SKP2, which is overexpressed in PCa^51^, plays a critical role in cancer development by controlling several cellular processes such as cell cycle regulation and cell proliferation, by degrading specific CDK inhibitors^52,53^. Overexpression of NUAK1 is associated with poor prognosis in many cancers, including colorectal, ovarian, and lung^54–56^. There is growing evidence showing NUAK1 is a target of multiple miRNAs, whose expression is frequently decreased during cancer progression to metastatic disease^57^. Additionally, NUAK1 is a positive regulator of cell cycle progression in breast cancer cells^58^. Our data supports the oncogenic function of SKP2 and NUAK1 in PCa, as their targeted degradation by miR-642a-5p resulted in cell cycle arrest. Conversely, RASSF3 functions as a tumor suppressor through stabilization of p53 and regulation of apoptosis and G1-S cell cycle arrest^59^, its downregulation increases malignant phenotypes of non-small cell lung cancer^60^, and is, in part, responsible for resistance to mammary tumor development in Neu transgenic mice^61^. These reports are contradictory to our observation of RASSF3 targeting by miR-642a-5p for inhibition of cell cycle progression in PCa, and coupled with the Oncomine TCGA data showing a higher level of RASSF3 in PCa tissue, suggests RASSF3 function may be dependent on cellular context. We found miR-642a-5p overexpression upregulated IGFBP3 and GPS2 expression. This data is consistent with previous reports; IGFBP3 overexpression has been shown to induce cell cycle arrest at G1 phase in breast cancer^62^ and suppress metastasis in PCa^63^, and GPS2 overexpression in osteocarcinoma was associated with cell cycle arrest^64^. Taken together, these data provide strong support for miR-642a-5p functioning as a potent tumor suppressor in PCa, an effect mediated by a coordinated change in expression of multiple targets leading to significant impact on cell cycle. We have previously studied other microRNAs, including miR-7-5p^65^ and miR-331-3p^24^, and identified multiple coordinately regulated downstream targets and signaling pathways, with a net effect of potent tumor inhibition, similar to what we identified herein.

There is an urgent need for new therapies for men with advanced PCa. Our data suggests miR-642a-5p is a potent PCa tumor suppressor in vitro and in vivo and that its successful replacement into PCa tissue could represent a new avenue of therapy for this disease. This is particularly relevant given that the field of RNA-based therapeutics is undergoing rapid change. With the recent approval of multiple siRNA drugs by the U.S. Food and Drug Administration there is an increased interest in using double stranded RNAs, including miRNAs, as therapies to treat human disease^15,66,67^. In that context, our data provides a foundation for further work to develop miR-642a-5p into an RNA-based PCa therapeutic.

## Methods

All the experimental protocols were performed in accordance with institutional guidelines and regulations of the Harry Perkins Institute of Medical Research.

### Cell culture, miRNA precursors, luciferase reporter constucts, siRNA molecules, small molecule inhibitors and cDNA expression constructs

22Rv1 and LNCaP PCa cells were obtained from the American Type Culture Collection (ATCC) and cultured at 37 °C/5% CO_2_ in RPMI-1640 supplemented with 10% fetal bovine serum (FBS). Synthetic miRNA molecules corresponding to human miR-642a-5p (hsa-miR-642a-5p; Cat #AM17100, Product ID: PM11477) and a negative control miRNA (miR-NC; Negative Control #1, Cat# AM17110) were sourced from Ambion (Thermo Fisher Scientific). The miRNA 3′UTR luciferase reporter constuct for WT1 (#Hmi T058379-MT06) was generated by GeneCopoeia (Rockville, MD). The miR-642a-5p perfect target and DOHH 3′UTR reporter constructs were generated by GenScript, Inc (Piscataway), as described^25^. Flexitube siRNAs to WT1 were from Qiagen (WT1#1 Cat#SI00008267; WT1#4 Cat#SI00008288; WT1#7 Cat#SI03056298; and WT1#8 Cat#SI03061331). The negative control siRNA (si-NC) was from Ambion (Cat#4390843). Tanespimycin (17-AAG) was from Selleckchem (Cat#S1141). Human WT1 cDNA (WT1-203, ENST00000379079.8, Ensembl) was synthesized (GenScript) and cloned into LeGO-iT2 lentiviral vector (a gift from Boris Fehse, Addgene plasmid # 27343).

### PCa cell xenograft model and tumor imaging

22Rv1 cells were transfected using Lipofectamine 2000 (Thermo Fisher Scientific) with 50 nM miR-642a-5p or miR-NC. At 72 h post transfection cells were trypsinized, counted, and 1.5 × 10^6^ cells in 150 µL of a 1:1 dilution of RPMI-1640 and Matrigel (BD BioSciences) was injected subcutaneously into male NSG mice (Animal Resource Centre, Western Australia) (10 per group). Generation of T2 weighted coronal and axial MRI images of NSG mice were by a 3.0 T MRS 3000 preclinical MRI system at the Australian Cancer Research Foundation Cancer Imaging Facility at the Harry Perkins Institute of Medical Research, Perth, Australia. All the experimental protocols were approved by the Harry Perkins Institute of Medical Research animal ethics committee (AE048/2016). All methods used for animal experimentation were carried out in accordance with the relevant guidelines and regulations of the Harry Perkins Institute of Medical Research Animal Ethics Committee. All animal work was carried out in compliance with the ARRIVE guidelines (http://www.nc3rs.org.uk/page.asp?id=1357).

### RNA-Sequencing expression profiling and analysis

For the RNA-Seq study, triplicate wells of 22Rv1 cells were transfected using Lipofectamine 2000 with 30 nM miR-642a-5p or miR-NC, and total RNA extracted from the samples 24 h post-transfection, using the Isolate II RNA kit (Bioline) according to the manufacturer’s instructions. The quantity and integrity of extracted RNA was determined using a 2100 Bioanalyzer (Agilent Technologies), before RNA-Seq analysis using the Illumina HiSeq 2500 at the Australian Genome Research Facility (AGRF; Victoria, Australia). Analysts at AGRF normalized the data with the R Bioconductor ‘EdgeR’ package (www.Bioconductor.org). Briefly, sequence counts were aligned to the genome, background corrected, log_2_ transformed, annotated, and a fold change analysis performed to compare treatment groups.

TargetScan (Version 7.2: March 2018) provided metadata on genes downregulated by miR-642a-5p in the RNA-Seq experiment. Gene Set Enrichment Analysis (GSEA) of the RNA-Seq data was performed as previously described^68^. The biological pathway targets of genes differentially expressed by miR-642a-5p were determined using Ingenuity Pathway Analysis (IPA, Ingenuity System, Inc. www.qiagenbioinformatics.com/products/ingenuity-pathway-analysis). The RNA-Seq data is available in the Gene Expression Omnibus under Accession Number GSE160736.

### Cell cycle analysis

22Rv1 or LNCaP PCa cells were transfected as described above with 30 nM miRNA (miR-642a-5p or miR-NC), or 20 nM siRNA (WT1 siRNA or si-NC) molecules. Following treatment for 72 h, floating and adherent cells were collected, fixed with cold 100% ethanol and stored at 4 °C. Fixed cells were stained with Propidium Iodide (PI) staining solution (25 µg/ml PI and 0.25 µg/ml RNase A in PBS), and analysed using the BD Accuri C6 Flow Cytometer and FlowJo Software (version 7.6.5), and the Dean-Jett-Fox method for gating cells.

### Annexin V-FITC/PI apoptosis assay

22Rv1 cells were transfected as described above with 30 nM miR-642a-5p or miR-NC for 72 h, or treated with 10 µM Camptothecin (Cayman Chemical) for 24 h (positive control for apoptosis). Apoptosis was measured using the Annexin V-FITC Apoptois Detection Kit I (BD Biosciences, NSW, Australia), using the manufacturer’s instructions. No stain, single stain and camptothecin treated cells were used to set gating strategies to identify live, apoptotic and dead cell populations. Samples were analysed using the BD Accuri C6 Flow Cytometer and software.

### Generation of 22Rv1 and LNCaP stably overexpressing WT1 cell lines

22Rv1 and LNCaP PCa cell lines stably expressing WT1-203 cDNA were generated by lentiviral transduction as previously described^69,70^. Briefly, 22Rv1 and LNCaP cells were infected with lentiviruses carrying LeGO-iT2-Empty or LeGO-iT2-WT1-203 plasmids, and transduced cells stably expressing tdTomato fluorescent protein were isolated by flow cytometry (FACSCalibur, BD Biosciences). The ectopic expression of WT1 in the isolated cells was validated with RT-qPCR.

### Transfection of miRNA precursors, siRNA molecules, cDNA overexpression constructs, and reporter gene assays

Parental 22Rv1 and LNCaP PCa cells, or 22Rv1 and LNCaP cells with stable expression of LeGO-iT2-WT1-203 or LeGO-iT2-Empty plasmids were seeded into 6-well plates or 10 cm diameter dishes and transfected as described above with miRNA or siRNA molecules at a final concentration of 10–30 nM. Cells were harvested 24 h post-transfection for RNA isolation and 3 days for protein extraction. For transient WT1 overexpression, 5 ng of LeGO-iT2-WT1-203 or LeGO-iT2-Empty plasmids were cotransfected with 10–30 nM miRNA molecules, and RNA and protein isolated at 2 and 3 days post-transfection, respectively.

For Luciferase reporter gene assays 22Rv1 and LNCaP cells were seeded into 6-well plates and co-transfected with 450 ng of firefly luciferase reporter plasmid DNA and 10 nM final concentration of either miR-642a-5p or miR-NC, using Lipofectamine 2000. After 48 h, lysates were assayed for firefly luciferase activity using the Luciferase Reporter Assay System (Promega) and a Fluostar OPTIMA microplate reader (BMG Labtech).

### Cell proliferation and colony forming assays

Parental or WT1-overexpressing 22Rv1 and LNCaP PCa cells were transfected in 10 cm dishes with miRNA molecules, siRNAs, or cDNA plasmid constructs (as described above). One day post-transfection, the cells were trypsinized and plated into 96 well plates, xCELLigence E-plates at 5000 cells/well, or into 10 cm dishes at 5000 cells (22Rv1) or 10,000 cells (LNCaP)/dish. Proliferation was evaluated in the 96 well plates at 1–7 days post-seeding using a CellTiter 96 AQ_ueous_ One Solution Cell Proliferation Assay (Promega) and the Fluostar OPTIMA microplate reader, according to the manufacturer’s instructions. Treatment with 17-AAG (1 µM) was 24 h post-seeding, and proliferation evaluated 1–7 days later using the same assay (Promega). The xCELLigence system (In Vitro Technologies) was used to measure the proliferation of cells in a real time setting for 72 h, according to the manufacturer’s instructions. The cells which were plated into the 10 cm dishes were assayed for colony formation after 2–3 weeks, using Crystal Violet staining as previously described^65^.

### RNA extraction, reverse transcription and quantitative polymerase chain reaction (RT-qPCR)

Total RNA was extracted from 22Rv1 and LNCaP PCa cells 24 h post-transfection with miRNAs or siRNAs, using TRIzol reagent as per the manufacturer’s instructions (Thermo Fisher Scientific). RNA was quantitated using the NanoDrop One spectrophotometer and 800 ng RNA was reverse transcribed using a QuantiTect reverse transcription kit (Qiagen). Quantitative PCR was performed in a Rotor-Gene Q thermocycler (Qiagen) using Bioline SensiMix (QT605-20) and validated QuantiTect primers (Qiagen) for HPRT1 (Cat#QT00059066, housekeeping control), WT1 (Cat#QT00059003), NUAK1 (Cat#QT00097447), RASSF3 (Cat#QT00051044), SKP2 (Cat#QT00006489), IGFBP3 (Cat#QT00072737), or GPS2 (Cat#QT00050715). For the WT1 overexpression experiments, the following primers from Sigma were used: WT1-exon8 (F) GTGACTTCAAGGACTGTGAACG; and WT1-exon9 (R) CGGGAGAACTTTCGCTGACAA.

Expression of target mRNAs relative to HPRT expression was calculated using the 2^−ΔΔCt^ method^71^.

### Protein extraction and western blotting

Protein extracts were prepared from cells lysed with mid-RIPA buffer and western blotting performed as described^24^. Briefly, protein samples were resolved in NuPAGE 4–12% Bis Tris gels (Thermo Fisher Scientific) and transferred to PVDF membranes (Roche). Membranes were blocked in Tris-buffered saline/Tween 20 (TBST)/5% skim milk and incubated with p21 (Cell signaling #2947S; 1:1000), p53 (Cell signaling #9282S; 1:500, or Santa Cruz #SC-126; 1:1000), or β-actin [AC-15] (Abcam ab6276; 1:5000) primary antibodies, followed by incubation with horseradish peroxidase (HRP) linked secondary antibodies [anti-mouse IgG (GE Healthcare; Cat#NA931V) or anti-rabbit IgG (GE Healthcare; Cat#NA934V)]. Protein detection was with enhanced chemiluminescence (ECL) using Luminata Classico Western HRP substrate (Millipore #WBLUC0100), and visualization was with either ECL-Hyperfilm (GE Healthcare; #GE HE28-9068-37) or the iBright Imaging System (Thermo Fisher Scientific).

### Clinical datasets

Oncomine (www.oncomine.org) analyses determined the differential expression levels of WT1, NUAK1, RASSF3, SKP2, IGFBP3, and GPS2 between normal and tumor prostate tissue cohorts from The Cancer Genome Atlas (TCGA), Tomlins, Taylors Prostate 3 or Yu Prostate data sets.

### Statistical analysis

Graphing and analysis of data was with GraphPad Prism 8 software. Use of the unpaired *t*-test (two-tailed) determined significant differences between clinical datasets in Oncomine, luciferase reporter assays, RT-qPCR assays, cell cycle analysis and apoptosis. Use of a two way ANOVA with repeated measures determined significant differences between PCa xenograft volumes. Log-rank (Mantel-Cox) and Gehan-Breslow-Wilcoxon testing was used for determining significant differences between xenograft survival curves. IPA molecular pathway analysis package used a Fisher’s exact test on genes identified by RNA-Seq.

## Supplementary Information

Supplementary Information.

## Abbreviations

miRNA: MicroRNA
PCa: Prostate cancer
RNA-Seq: RNA-sequencing
3′UTR: 3′Untranslated region
siRNA: Small interfering RNA
RT-qPCR: Reverse transcription and quantitative polymerase chain reaction
DOHH: Deoxyhypusine hydroxylase
WT1: Wilms Tumor 1 gene
NUAK1: NUAK family SNF1-like kinase 1
RASSF3: Ras association domain family member 3
SKP2: S-phase kinase-associated protein 2
GPS2: G Protein Pathway Suppressor 2
IGFBP3: Insulin-like growth factor-binding protein 3
17-AAG: 17-(Allylamino)-17-demethoxygeldanamycin (Tanespimycin)
