# Supplementary material for: The tumor suppressor miR-642a-5p targets Wilms Tumor 1 gene and cell-cycle progression in prostate cancer
Source: Sci Rep. 2021 Sep 9;11:18003. doi: 10.1038/s41598-021-97190-x (PMC8429423; doi:10.1038/s41598-021-97190-x)
Supplement: Supplementary file 1 — Supplementary Information. [file 41598_2021_97190_MOESM1_ESM.pdf]

The tumor suppressor miR-642a-5p targets Wilms Tumor 1 gene and cell-cycle progression in prostate cancer

Supplementary Tables and Figure Legends

Supplementary Table S1. Downregulated miR-642a-5p target genes compiled from RNA-Seq data. Stratification of genes is in descending order for ≥-0.5 $\log_2$ fold change ($p<0.05$).

Supplementary Table S2. Upregulated miR-642a-5p target genes compiled from RNA-Seq data. Stratification of genes is in descending order for ≥0.5 $\log_2$ fold change ($p<0.05$).

Supplementary Table S3. Downregulated miR-642a-5p target genes compiled from RNA-Seq data. Stratification of genes is in descending order for the total number of miR-642a-5p sites within their 3′UTR (TargetScan 7.2), together with a ≥0.5 $\log_2$ fold change. ^^ denotes published target. Genes that are in bolded text and denoted with ** are those studied herein.

Supplementary Table S4. Upregulated miR-642a-5p target genes compiled from RNA-Seq data. Stratification of genes is in descending order for the total number of miR-642a-5p sites within their 3′UTR (TargetScan 7.2), together with a ≥0.5 $\log_2$ fold change.

Supplementary Figure S1. Original western blots of miR-642a-5p or WT1 siRNA treated 22Rv1 and LNCaP cells. (A) Western blot analysis of p21 and p53 protein expression 72 h post-transfection of 22Rv1 cells with 30 nM miR-642a-5p or miR-NC. β-actin is the loading control. The blots shown here are the original non-cropped blots used to generate Figure 2E, with the red boxes indicating the lanes used. **(B)** Western blot analysis of p21 protein expression 72 h post-transfection of 22Rv1 or LNCaP cells with 20 nM WT1 siRNA or si-NC. β-actin is the loading control. The blots shown here are the original non-cropped blots used to generate Figure 5E, with the red boxes indicating the lanes used. **(C)** Western blot analysis of p53 protein expression 72 h post-transfection of 22Rv1 cells with 20 nM WT1 siRNA or si-NC. β-actin is the loading control. The blots shown here are the original non-cropped blots used to generate Figure 5E, with the red boxes indicating the lanes used, and two different exposures of p53 are included.

Supplementary Figure S2. RT-qPCR validation of siRNA mediated knockdown of WT1 gene expression. (A) 22Rv1 cells were transfected with 20 nM of four different WT1 siRNA constructs or a negative control siRNA (si-NC). RNA extraction and RT-qPCR was at 24 h post transfection. Error bars = SD, *p<0.05 and **p<0.005 relative to si-NC. **(B-E)** 22Rv1 and LNCaP cells were transfected with WT1#8 siRNA or si-NC for functional assays reported in Figure 5. RNA extraction was at 24 h post transfection, and RT-qPCR analysis of WT1 expression is normalized to HPRT housekeeping gene expression, calculated using the $2^{-\Delta\Delta Ct}$ method, and relative to si-NC. Error bars = SD; *p<0.05, **p<0.005 relative to si-NC.

Supplementary Figure 1

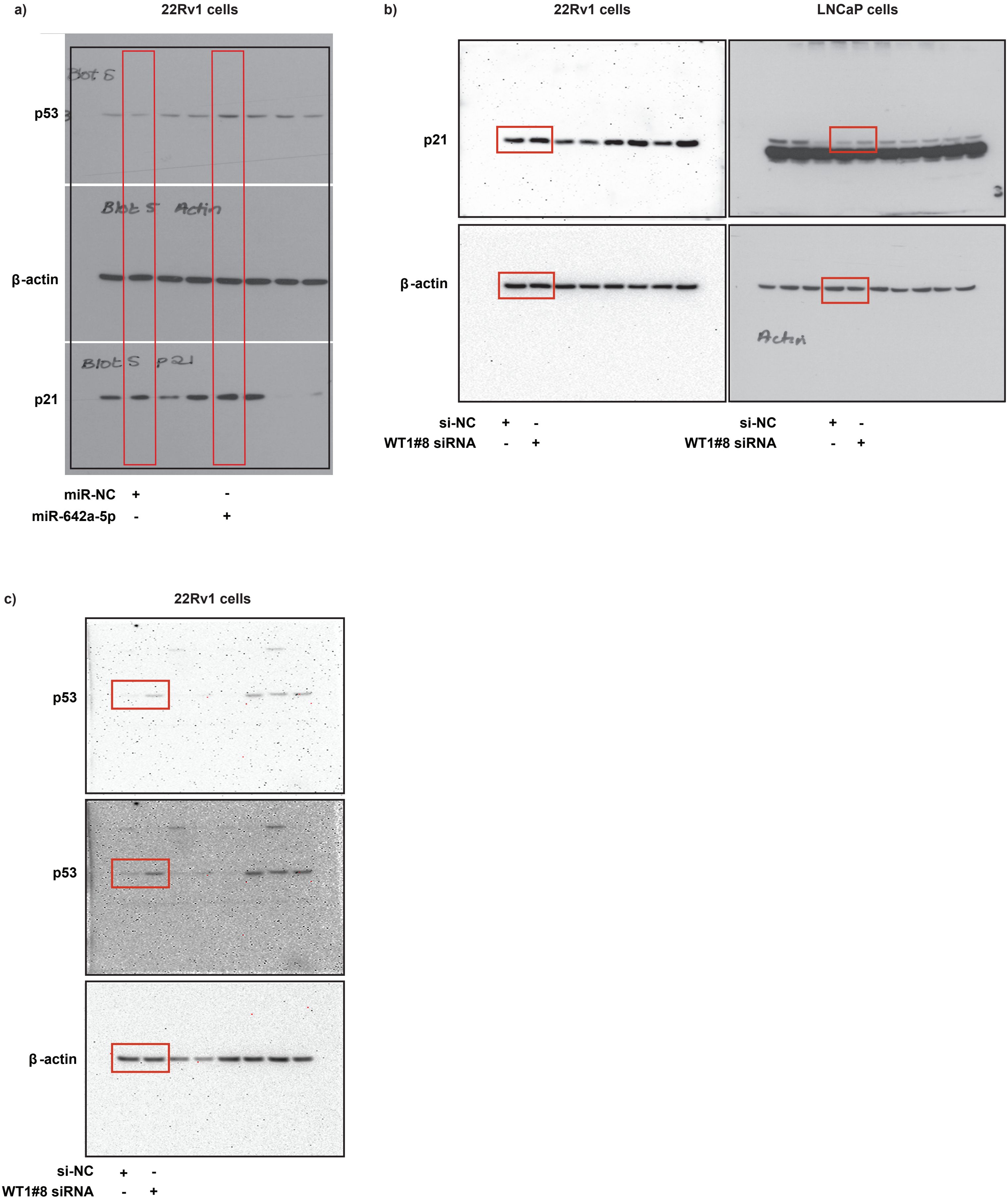

Supplementary Figure 2

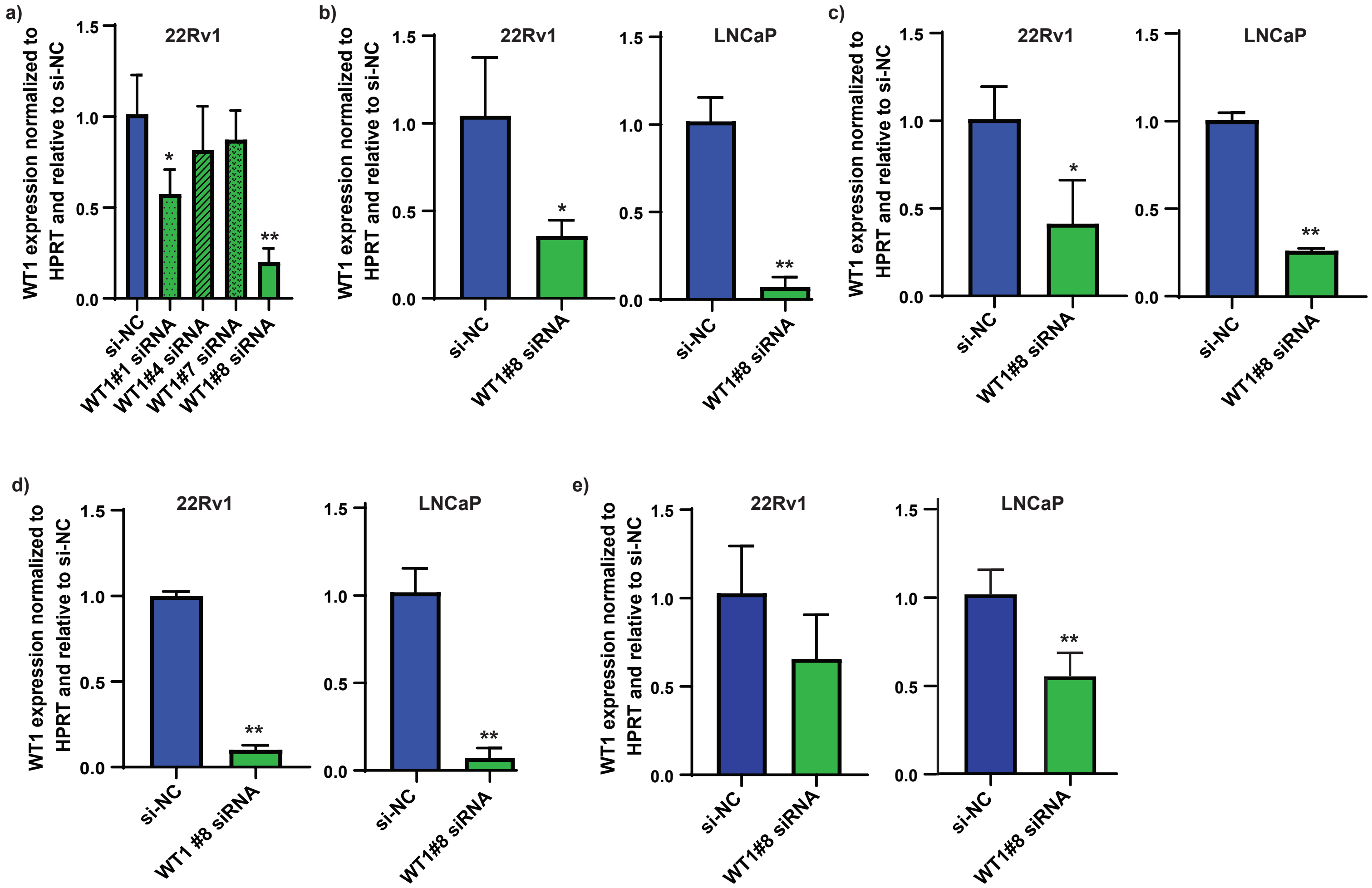

Supplementary Table S1.

Gene	Gene Description	Ensemble Gene ID	$\log_2$ FC
RP11-386G11.10		ENSG00000258017	-1.37
MCF2L	MCF.2 cell line derived transforming sequence like	ENSG00000126217	-1.33
EIF3C	eukaryotic translation initiation factor 3 subunit C	ENSG00000184110	-1.26
DQX1	DEAQ-box RNA dependent ATPase 1	ENSG00000144045	-1.08
AC002467.7		ENSG00000241764	-1.07
RP11-67L2.2		ENSG00000273033	-1.06
ILF2P1	interleukin enhancer binding factor 2 pseudogene 1	ENSG00000244226	-1.01
RPL39P3	ribosomal protein L39 pseudogene 3	ENSG00000235174	-0.99
RN7SKP291	RNA, 7SK small nuclear pseudogene 291	ENSG00000199831	-0.95
MEF2C	myocyte enhancer factor 2C	ENSG00000081189	-0.95
FAM133B	family with sequence similarity 133 member B	ENSG00000234545	-0.93
SNORA80B	small nucleolar RNA, H/ACA box 80B	ENSG00000206633	-0.93
LAMP1	lysosomal associated membrane protein 1	ENSG00000185896	-0.92
MYO1A	myosin IA	ENSG00000166866	-0.91
RP11-120D5.1		ENSG00000234129	-0.91
NECAP1P1	NECAP endocytosis associated 1 pseudogene 1	ENSG00000178503	-0.90
SNORD3A	small nucleolar RNA, C/D box 3A	ENSG00000263934	-0.89
CCT6B	chaperonin containing TCP1 subunit 6B	ENSG00000132141	-0.88
GCOM2	GRINL1B complex locus 2 (pseudogene)	ENSG00000227725	-0.88
USP12	ubiquitin specific peptidase 12	ENSG00000152484	-0.87
DIRC2	disrupted in renal carcinoma 2	ENSG00000138463	-0.85
PAPSS2	3'-phosphoadenosine 5'-phosphosulfate synthase 2	ENSG00000198682	-0.85
PROCA1	protein interacting with cyclin A1	ENSG00000167525	-0.84
UBBP1	ubiquitin B pseudogene 1	ENSG00000230037	-0.84
NQO1	NAD(P)H quinone dehydrogenase 1	ENSG00000181019	-0.83
RP11-79P5.10			-0.82
SNORA71D	small nucleolar RNA, H/ACA box 71D	ENSG00000200354	-0.81
BAK1P1	BCL2 antagonist/killer 1 pseudogene 1	ENSG00000175730	-0.80
FRG1BP	FSHD region gene 1 family member B, pseudogene	ENSG00000149531	-0.80
STAC	SH3 and cysteine rich domain	ENSG00000144681	-0.80
RNU1-67P	RNA, U1 small nuclear 67, pseudogene	ENSG00000207175	-0.79
RP11-174G6.5		ENSG00000261324	-0.79
IRF1	interferon regulatory factor 1	ENSG00000125347	-0.79
TIMM8BP2	translocase of inner mitochondrial membrane 8B pseudogene 2	ENSG00000224908	-0.79
RP3-510O8.3		ENSG00000220734	-0.78
AAK1	AP2 associated kinase 1	ENSG00000115977	-0.78
PRR26	proline rich 26	ENSG00000180525	-0.77
RP11-545I5.3		ENSG00000235652	-0.77
RBMS2	RNA binding motif single stranded interacting protein 2	ENSG00000076067	-0.77
TLE6	transducin like enhancer of split 6	ENSG00000104953	-0.77
RNU5F-1	RNA, U5F small nuclear 1	ENSG00000199377	-0.76

MEF2D	myocyte enhancer factor 2D	ENSG00000116604	-0.76
NECAB1	N-terminal EF-hand calcium binding protein 1	ENSG00000123119	-0.76
NUAK1	NUAK family kinase 1	ENSG00000074590	-0.76
RP3-359N14.1		ENSG00000219088	-0.75
RP11-296O14.3		ENSG00000203739	-0.75
RPL38P3	ribosomal protein L38 pseudogene 3	ENSG00000244422	-0.75
IGSF3	immunoglobulin superfamily member 3	ENSG00000143061	-0.75
CTD-2102P23.1		ENSG00000255875	-0.74
RAB6A	RAB6A, member RAS oncogene family	ENSG00000175582	-0.74
CASC4P1	cancer susceptibility candidate 4 pseudogene 1	ENSG00000238132	-0.73
TYMSOS	TYMS opposite strand	ENSG00000176912	-0.73
AC010878.3		ENSG00000225402	-0.71
C4orf3	chromosome 4 open reading frame 3	ENSG00000164096	-0.71
CMTM4	CKLF like MARVEL transmembrane domain containing 4	ENSG00000183723	-0.71
RAD9B	RAD9 checkpoint clamp component B	ENSG00000151164	-0.71
AC006978.6		ENSG00000235859	-0.71
RP11-101E13.5		ENSG00000272579	-0.70
SPINT2	serine peptidase inhibitor, Kunitz type 2	ENSG00000167642	-0.70
BZW1P1	basic leucine zipper and W2 domains 1 pseudogene 1	ENSG00000236686	-0.70
SNORA2C	small nucleolar RNA, H/ACA box 2C	ENSG00000221491	-0.70
TSPAN15	tetraspanin 15	ENSG00000099282	-0.70
RP1-228P16.7		ENSG00000226138	-0.70
ADPRHL1	ADP-ribosylhydrolase like 1	ENSG00000153531	-0.70
SEPT_3	septin 3	ENSG00000100167	-0.70
APLF	aprataxin and PNKP like factor	ENSG00000169621	-0.70
STRAP	serine/threonine kinase receptor associated protein	ENSG00000023734	-0.69
DOHH	deoxyhypusine hydroxylase/monooxygenase	ENSG00000129932	-0.69
RP11-651P23.4		ENSG00000224831	-0.69
SLC16A6	solute carrier family 16 member 6	ENSG00000108932	-0.69
DR1	down-regulator of transcription 1	ENSG00000117505	-0.68
NECAP1	NECAP endocytosis associated 1	ENSG00000089818	-0.66
SNORA5C	small nucleolar RNA, H/ACA box 5C	ENSG00000201772	-0.65
DMBX1	diencephalon/mesencephalon homeobox 1	ENSG00000197587	-0.65
PCNP	PEST proteolytic signal containing nuclear protein	ENSG00000081154	-0.65
AOX1	aldehyde oxidase 1	ENSG00000138356	-0.64
MANBA	mannosidase beta	ENSG00000109323	-0.64
CERS2	ceramide synthase 2	ENSG00000143418	-0.64
BZW1P2	basic leucine zipper and W2 domains 1 pseudogene 2	ENSG00000198406	-0.63
TTC7B	tetratricopeptide repeat domain 7B	ENSG00000165914	-0.63
RGS1	regulator of G-protein signaling 1	ENSG00000090104	-0.63
PCYOX1L	prenylcysteine oxidase 1 like	ENSG00000145882	-0.63
ZNF610	zinc finger protein 610	ENSG00000167554	-0.63

RPS15AP38	ribosomal protein S15a pseudogene 38	ENSG00000237668	-0.62
CDK7PS			-0.62
FAM134C	family with sequence similarity 134 member C	ENSG00000141699	-0.62
SUV39H1	suppressor of variegation 3-9 homolog 1	ENSG00000101945	-0.62
PRKAA2	protein kinase AMP-activated catalytic subunit alpha 2	ENSG00000162409	-0.62
RP11-1017G21.5		ENSG00000271780	-0.62
ZFAND6	zinc finger AN1-type containing 6	ENSG00000086666	-0.62
NCK2	NCK adaptor protein 2	ENSG00000071051	-0.62
CASC4	cancer susceptibility candidate 4	ENSG00000166734	-0.61
NCBP2	nuclear cap binding protein subunit 2	ENSG00000114503	-0.61
MFHAS1	malignant fibrous histiocytoma amplified sequence 1	ENSG00000147324	-0.61
PRDX2P1	peroxiredoxin 2 pseudogene 1	ENSG00000215049	-0.61
SNX10	sorting nexin 10	ENSG00000086300	-0.61
RMDN3	regulator of microtubule dynamics 3	ENSG00000137824	-0.60
NUDT21	nudix hydrolase 21	ENSG00000167005	-0.60
ARF4-AS1	ARF4 antisense RNA 1	ENSG00000272146	-0.60
TMEM183A	transmembrane protein 183A	ENSG00000163444	-0.60
RP11-18B3.2		ENSG00000232486	-0.60
ACBD7	acyl-CoA binding domain containing 7	ENSG00000176244	-0.60
AIFM2	apoptosis inducing factor, mitochondria associated 2	ENSG00000042286	-0.60
MKRN2	makorin ring finger protein 2	ENSG00000075975	-0.60
CTD-2206N4.4		ENSG00000263818	-0.59
STYK1	serine/threonine/tyrosine kinase 1	ENSG00000060140	-0.59
UGT2B11	UDP glucuronosyltransferase family 2 member B11	ENSG00000213759	-0.59
CBX5P1	chromobox 5 pseudogene 1	ENSG00000241535	-0.59
RP11-134G8.7		ENSG00000224536	-0.59
KHDC1	KH homology domain containing 1	ENSG00000135314	-0.58
RPL7AP34	ribosomal protein L7a pseudogene 34	ENSG00000213312	-0.58
ZNF354C	zinc finger protein 354C	ENSG00000177932	-0.58
ZNF674-AS1	ZNF674 antisense RNA 1 (head to head)	ENSG00000230844	-0.58
RHOBTB3	Rho related BTB domain containing 3	ENSG00000164292	-0.58
RPL7P9	ribosomal protein L7 pseudogene 9	ENSG00000137970	-0.58
PLEKHM3	pleckstrin homology domain containing M3	ENSG00000178385	-0.58
LARS2	leucyl-tRNA synthetase 2, mitochondrial	ENSG00000011376	-0.57
KLHL23	kelch like family member 23	ENSG00000213160	-0.57
FUZ	fuzzy planar cell polarity protein	ENSG00000010361	-0.57
NOV	nephroblastoma overexpressed	ENSG00000136999	-0.57
FXN	frataxin	ENSG00000165060	-0.57
WTH3DI	RAB6C-like	ENSG00000233087	-0.57
FAM120C	family with sequence similarity 120C	ENSG00000184083	-0.56
SLC7A2	solute carrier family 7 member 2	ENSG00000003989	-0.56
APMAP	adipocyte plasma membrane associated protein	ENSG00000101474	-0.56
TP53INP2	tumor protein p53 inducible nuclear protein 2	ENSG00000078804	-0.56
RRBP1	ribosome binding protein 1	ENSG00000125844	-0.56

RTN4RL1	reticulon 4 receptor like 1	ENSG00000185924	-0.56
WT1	Wilms tumor 1	ENSG00000184937	-0.55
ZEB1-AS1	ZEB1 antisense RNA 1	ENSG00000237036	-0.55
HABP2	hyaluronan binding protein 2	ENSG00000148702	-0.55
ADRA1A	adrenoceptor alpha 1A	ENSG00000120907	-0.55
P2RX6	purinergic receptor P2X 6	ENSG00000099957	-0.55
ABHD10	abhydrolase domain containing 10	ENSG00000144827	-0.55
SMG1P3	SMG1P3, nonsense mediated mRNA decay associated PI3K related kinase pseudogene 3	ENSG00000180747	-0.54
CCDC102B	coiled-coil domain containing 102B	ENSG00000150636	-0.54
SCARNA22	small Cajal body-specific RNA 22	ENSG00000249784	-0.54
RASSF3	Ras association domain family member 3	ENSG00000153179	-0.54
CD2AP	CD2 associated protein	ENSG00000198087	-0.54
PGGT1B	protein geranylgeranyltransferase type I subunit beta	ENSG00000164219	-0.54
EIF5P1	eukaryotic translation initiation factor 5 pseudogene 1	ENSG00000215319	-0.54
CTD-2201E18.3		ENSG00000177738	-0.53
FOXN2	forkhead box N2	ENSG00000170802	-0.53
PPP3CA	protein phosphatase 3 catalytic subunit alpha	ENSG00000138814	-0.53
SIRT5	sirtuin 5	ENSG00000124523	-0.53
AC010132.11		ENSG00000234983	-0.53
CARNMT1	carnosine N-methyltransferase 1	ENSG00000156017	-0.53
IGHMBP2	immunoglobulin mu binding protein 2	ENSG00000132740	-0.53
FGF13	fibroblast growth factor 13	ENSG00000129682	-0.52
RP11-368I7.4		ENSG00000260259	-0.52
MORF4L1P1	mortality factor 4 like 1 pseudogene 1	ENSG00000218283	-0.52
CDADC1	cytidine and dCMP deaminase domain containing 1	ENSG00000102543	-0.52
KLF12	Kruppel like factor 12	ENSG00000118922	-0.52
ARPIN	actin-related protein 2/3 complex inhibitor	ENSG00000242498	-0.52
CDC37L1	cell division cycle 37 like 1	ENSG00000106993	-0.52
TSPAN8	tetraspanin 8	ENSG00000127324	-0.52
ZDHHC5	zinc finger DHHC-type containing 5	ENSG00000156599	-0.52
CELF3	CUGBP, Elav-like family member 3	ENSG00000159409	-0.52
ANAPC13	anaphase promoting complex subunit 13	ENSG00000129055	-0.52
LDHAP2	lactate dehydrogenase A pseudogene 2	ENSG00000235674	-0.51
MRPL20	mitochondrial ribosomal protein L20	ENSG00000242485	-0.51
BZW1	basic leucine zipper and W2 domains 1	ENSG00000082153	-0.51
MEX3A	mex-3 RNA binding family member A	ENSG00000254726	-0.51
ZNF576	zinc finger protein 576	ENSG00000124444	-0.51
NHLRC3	NHL repeat containing 3	ENSG00000188811	-0.51
DCAF16	DDB1 and CUL4 associated factor 16	ENSG00000163257	-0.51
AMD1P3	adenosylmethionine decarboxylase 1 pseudogene 3	ENSG00000249286	-0.51
ZBED5-AS1	ZBED5 antisense RNA 1	ENSG00000247271	-0.51
NDUFA5P11	NADH:ubiquinone oxidoreductase subunit A5 pseudogene 11	ENSG00000259918	-0.51
RP11-9E17.1		ENSG00000277534	-0.50

SKP2	S-phase kinase associated protein 2	ENSG00000145604	-0.50
YBX1P2	Y-box binding protein 1 pseudogene 2	ENSG00000231167	-0.50
TMEM123	transmembrane protein 123	ENSG00000152558	-0.50
TMTC2	transmembrane and tetratricopeptide repeat containing 2	ENSG00000179104	-0.50
DDC	dopa decarboxylase	ENSG00000132437	-0.50
POLR2M	RNA polymerase II subunit M	ENSG00000255529	-0.50

Supplementary Table S2.

Gene	Gene Description	Ensemble Gene ID	log_2 FC
MKX	mohawk homeobox	ENSG00000150051	1.33
DNAH7	dynein axonemal heavy chain 7	ENSG00000118997	1.24
SLC17A7	solute carrier family 17 member 7	ENSG00000104888	1.23
CRIP1	cysteine rich protein 1	ENSG00000213145	1.22
SYCE1L	synaptonemal complex central element protein 1 like	ENSG00000205078	1.18
UBXN10	UBX domain protein 10	ENSG00000162543	1.18
CYP4F8	cytochrome P450 family 4 subfamily F member 8	ENSG00000186526	1.17
GIPC3	GIPC PDZ domain containing family member 3	ENSG00000179855	1.10
CTD-2124B8.2		ENSG00000270558	1.08
RP11-488L18.1		ENSG00000214144	1.06
RP11-863K10.4		ENSG00000233170	1.05
CRLF3	cytokine receptor like factor 3	ENSG00000176390	1.05
CAMKV	CaM kinase like vesicle associated	ENSG00000164076	1.02
ITGB8	integrin subunit beta 8	ENSG00000105855	1.00
RP11-20I23.2		ENSG00000279520	0.97
FGFR4	fibroblast growth factor receptor 4	ENSG00000160867	0.97
TTC16	tetratricopeptide repeat domain 16	ENSG00000167094	0.95
ZMYND10	zinc finger MYND-type containing 10	ENSG00000004838	0.94
PTP4A1P1	protein tyrosine phosphatase type IVA, member 1 pseudogene 1	ENSG00000231158	0.93
MAP6D1	MAP6 domain containing 1	ENSG00000180834	0.93
CALCB	calcitonin related polypeptide beta	ENSG00000175868	0.93
RGS16	regulator of G-protein signaling 16	ENSG00000143333	0.92
HSPG2	heparan sulfate proteoglycan 2	ENSG00000142798	0.92
CADM2	cell adhesion molecule 2	ENSG00000175161	0.91
TTC21A	tetratricopeptide repeat domain 21A	ENSG00000168026	0.89
LRRC26	leucine rich repeat containing 26	ENSG00000184709	0.89
MFAP3L	microfibrillar associated protein 3 like	ENSG00000198948	0.88
RP3-412A9.17		ENSG00000278920	0.88
ZNF788	zinc finger family member 788	ENSG00000214189	0.88
CITED4	Cbp/p300 interacting transactivator with Glu/Asp rich carboxy-terminal domain 4	ENSG00000179862	0.88
SNRPC	small nuclear ribonucleoprotein polypeptide C	ENSG00000124562	0.88
SPSB1	splA/ryanodine receptor domain and SOCS box containing 1	ENSG00000171621	0.88
KAZN	kazrin, periplakin interacting protein	ENSG00000189337	0.88
RP11-399K21.14		ENSG00000272692	0.87
RSL24D1P11	ribosomal L24 domain containing 1 pseudogene 11	ENSG00000231544	0.87
CNIH3	cornichon family AMPA receptor auxiliary protein 3	ENSG00000143786	0.87
TMEM158	transmembrane protein 158 (gene/pseudogene)	ENSG00000249992	0.86
NDUFB8P2	NADH:ubiquinone oxidoreductase subunit B8 pseudogene 2	ENSG00000270264	0.86

ANP32BP1	acidic nuclear phosphoprotein 32 family member B pseudogene 1	ENSG00000259790	0.85
SYCE3	synaptonemal complex central element protein 3	ENSG00000217442	0.85
COMMD10	COMM domain containing 10	ENSG00000145781	0.85
ZBTB18	zinc finger and BTB domain containing 18	ENSG00000179456	0.85
RP4-736I12.1		ENSG00000225923	0.84
ADCY1	adenylate cyclase 1	ENSG00000164742	0.83
CREBRF	CREB3 regulatory factor	ENSG00000164463	0.83
DENND6A	DENN domain containing 6A	ENSG00000174839	0.82
RP3-467N11.2		ENSG00000270987	0.82
KLRD1	killer cell lectin like receptor D1	ENSG00000134539	0.81
NPTX1	neuronal pentraxin 1	ENSG00000171246	0.81
TRIP10	thyroid hormone receptor interactor 10	ENSG00000125733	0.81
ITGA6	integrin subunit alpha 6	ENSG00000091409	0.80
CELSR2	cadherin EGF LAG seven-pass G-type receptor 2	ENSG00000143126	0.80
SPATA6	spermatogenesis associated 6	ENSG00000132122	0.79
DUSP9	dual specificity phosphatase 9	ENSG00000130829	0.79
IGFBP3	insulin like growth factor binding protein 3	ENSG00000146674	0.79
GGACT	gamma-glutamylamine cyclotransferase	ENSG00000134864	0.79
RP11-435F17.3		ENSG00000241889	0.79
MESP1	mesoderm posterior bHLH transcription factor 1	ENSG00000166823	0.78
DLX2	distal-less homeobox 2	ENSG00000115844	0.78
TYRO3P	TYRO3P protein tyrosine kinase pseudogene	ENSG00000259581	0.78
STPG3	sperm-tail PG-rich repeat containing 3	ENSG00000197768	0.78
STAG3L2	stromal antigen 3-like 2 (pseudogene)	ENSG00000277072	0.78
PLCD1	phospholipase C delta 1	ENSG00000187091	0.77
RP11-752G15.4		ENSG00000259707	0.77
HCCS	holocytochrome c synthase	ENSG00000004961	0.77
SPOPL	speckle type BTB/POZ protein like	ENSG00000144228	0.77
RLN2	relaxin 2	ENSG00000107014	0.77
MMP25-AS1	MMP25 antisense RNA 1	ENSG00000261971	0.77
SEPHS1P6	selenophosphate synthetase 1 pseudogene 6	ENSG00000213938	0.76
RP11-701H24.7		ENSG00000271347	0.76
CHRM4	cholinergic receptor muscarinic 4	ENSG00000180720	0.76
ZFHX2	zinc finger homeobox 2	ENSG00000136367	0.76
NOP56P3	NOP56 ribonucleoprotein pseudogene 3	ENSG00000257956	0.75
POLR3G	RNA polymerase III subunit G	ENSG00000113356	0.75
C5orf51	chromosome 5 open reading frame 51	ENSG00000205765	0.75
PPP1R2P3	protein phosphatase 1 regulatory inhibitor subunit 2 pseudogene 3	ENSG00000231989	0.75
EMC10	ER membrane protein complex subunit 10	ENSG00000161671	0.74
SLC4A8	solute carrier family 4 member 8	ENSG00000050438	0.74
IZUMO4	IZUMO family member 4	ENSG00000099840	0.74

MAPRE1P1	MAPRE1 pseudogene 1	ENSG00000253915	0.74
DDN	dendrin	ENSG00000181418	0.74
TTC39B	tetratricopeptide repeat domain 39B	ENSG00000155158	0.74
CCDC85B	coiled-coil domain containing 85B	ENSG00000175602	0.73
CFAP69	cilia and flagella associated protein 69	ENSG00000105792	0.73
C9orf116	chromosome 9 open reading frame 116	ENSG00000160345	0.73
RP11-158K1.3		ENSG00000253106	0.73
CARMIL2	capping protein regulator and myosin 1 linker 2	ENSG00000159753	0.73
GPS2	G protein pathway suppressor 2	ENSG00000132522	0.73
DUSP18	dual specificity phosphatase 18	ENSG00000167065	0.73
SLC16A9	solute carrier family 16 member 9	ENSG00000165449	0.73
MMP17	matrix metallopeptidase 17	ENSG00000198598	0.72
KDELC1	KDEL motif containing 1	ENSG00000134901	0.72
TLL1	tolloid like 1	ENSG00000038295	0.72
SLC52A1	solute carrier family 52 member 1	ENSG00000132517	0.72
UBE2V1	ubiquitin conjugating enzyme E2 V1	ENSG00000244687	0.72
RP11-317N8.4		ENSG00000257307	0.72
GFPT2	glutamine-fructose-6-phosphate transaminase 2	ENSG00000131459	0.72
CHPF	chondroitin polymerizing factor	ENSG00000123989	0.72
RP11-204C16.4		ENSG00000217624	0.72
PALD1	phosphatase domain containing, paladin 1	ENSG00000107719	0.72
UBTD1	ubiquitin domain containing 1	ENSG00000165886	0.71
TMEM121	transmembrane protein 121	ENSG00000184986	0.71
ERLIN1	ER lipid raft associated 1	ENSG00000107566	0.71
RP13-444K19.1		ENSG00000235916	0.71
DTX3L	deltex E3 ubiquitin ligase 3L	ENSG00000163840	0.70
YIPF1	Yip1 domain family member 1	ENSG00000058799	0.70
CEBPA-AS1	CEBPA antisense RNA 1 (head to head)	ENSG00000267296	0.70
AC141586.5		ENSG00000215154	0.70
RDX	radixin	ENSG00000137710	0.70
DDX3Y	DEAD-box helicase 3, Y-linked	ENSG00000067048	0.70
MAGEA12	MAGE family member A12	ENSG00000213401	0.69
COL6A2	collagen type VI alpha 2 chain	ENSG00000142173	0.69
ITGA5	integrin subunit alpha 5	ENSG00000161638	0.69
PNRC2P1	proline rich nuclear receptor coactivator 2 pseudogene 1	ENSG00000228217	0.68
BRIX1P1	BRX1, biogenesis of ribosomes pseudogene 1	ENSG00000254025	0.68
RGS14	regulator of G-protein signaling 14	ENSG00000169220	0.68
PRR7	proline rich 7, synaptic	ENSG00000131188	0.67
SNRPCP2	small nuclear ribonucleoprotein polypeptide C pseudogene 2	ENSG00000251235	0.67
RP5-1053E7.3		ENSG00000214812	0.67
SHANK3	SH3 and multiple ankyrin repeat domains 3	ENSG00000283243	0.67
GPX7	glutathione peroxidase 7	ENSG00000116157	0.67
KLRG2	killer cell lectin like receptor G2	ENSG00000188883	0.67

PMM2	phosphomannomutase 2	ENSG00000140650	0.66
ACVR2A	activin A receptor type 2A	ENSG00000121989	0.66
NARF	nuclear prelamin A recognition factor	ENSG00000141562	0.66
ARL8B	ADP ribosylation factor like GTPase 8B	ENSG00000134108	0.66
PROSC	proline synthetase cotranscribed homolog (bacterial)	ENSG00000147471	0.66
SLC30A6	solute carrier family 30 member 6	ENSG00000152683	0.66
CTB-158E9.2		ENSG00000253886	0.65
STAT3	signal transducer and activator of transcription 3	ENSG00000168610	0.65
AC013470.6		ENSG00000236048	0.65
FSCN2	fascin actin-bundling protein 2, retinal	ENSG00000186765	0.65
USP25	ubiquitin specific peptidase 25	ENSG00000155313	0.65
LLPH	LLP homolog, long-term synaptic facilitation	ENSG00000139233	0.65
RP11-813P10.2		ENSG00000237176	0.64
C8orf58	chromosome 8 open reading frame 58	ENSG00000241852	0.64
MEMO1	mediator of cell motility 1	ENSG00000162959	0.64
ZXDA	zinc finger, X-linked, duplicated A	ENSG00000198205	0.64
PDCD4	programmed cell death 4 (neoplastic transformation inhibitor)	ENSG00000150593	0.64
ZFP36	ZFP36 ring finger protein	ENSG00000128016	0.64
RELL1	RELT like 1	ENSG00000181826	0.64
ZNF225	zinc finger protein 225	ENSG00000256294	0.63
FAM86C1	family with sequence similarity 86 member C1	ENSG00000158483	0.63
SLC45A4	solute carrier family 45 member 4	ENSG00000022567	0.63
AREG	amphiregulin	ENSG00000109321	0.63
KLHL2	kelch like family member 2	ENSG00000109466	0.63
SEMA3G	semaphorin 3G	ENSG00000010319	0.63
DEPDC4	DEP domain containing 4	ENSG00000166153	0.63
GXYLT2	glucoside xylosyltransferase 2	ENSG00000172986	0.63
UBLCP1	ubiquitin like domain containing CTD phosphatase 1	ENSG00000164332	0.62
ULK4	unc-51 like kinase 4	ENSG00000168038	0.62
HELB	DNA helicase B	ENSG00000127311	0.62
SERBP1P6	SERPINE1 mRNA binding protein 1 pseudogene 6	ENSG00000248873	0.62
IQCB2P	IQ motif containing B2 pseudogene	ENSG00000217539	0.62
DDIT4	DNA damage inducible transcript 4	ENSG00000168209	0.62
SHC2	SHC adaptor protein 2	ENSG00000129946	0.62
FER1L4	fer-1 like family member 4, pseudogene	ENSG00000088340	0.61
TERT	telomerase reverse transcriptase	ENSG00000164362	0.61
DGKE	diacylglycerol kinase epsilon	ENSG00000153933	0.61
TEX15	testis expressed 15	ENSG00000133863	0.61
CFAP43	cilia and flagella associated protein 43	ENSG00000197748	0.61
CXorf56	chromosome X open reading frame 56	ENSG00000018610	0.61
PRSS41	protease, serine 41	ENSG00000215148	0.61
ZFPM1	zinc finger protein, FOG family member 1	ENSG00000179588	0.61
OTUD4P1	OTUD4 pseudogene 1	ENSG00000118976	0.60
ALPK1	alpha kinase 1	ENSG00000073331	0.60

RP11-568K15.1		ENSG00000242193	0.60
CTU1	cytosolic thiouridylase subunit 1	ENSG00000142544	0.59
FGFR1	fibroblast growth factor receptor 1	ENSG00000077782	0.59
HOXB6	homeobox B6	ENSG00000108511	0.59
CECR2	CECR2, histone acetyl-lysine reader	ENSG00000099954	0.59
RNF166	ring finger protein 166	ENSG00000158717	0.59
ARPC4	actin related protein 2/3 complex subunit 4	ENSG00000241553	0.59
TDGP1	thymine-DNA glycosylase pseudogene 1	ENSG00000255725	0.59
GALC	galactosylceramidase	ENSG00000054983	0.59
FNIP2	folliculin interacting protein 2	ENSG00000052795	0.59
FOXP1-IT1	FOXP1 intronic transcript 1	ENSG00000242094	0.59
RPS6KA2	ribosomal protein S6 kinase A2	ENSG00000071242	0.59
ZNF365	zinc finger protein 365	ENSG00000138311	0.58
SLC8B1	solute carrier family 8 member B1	ENSG00000089060	0.58
EVA1B	eva-1 homolog B	ENSG00000142694	0.58
ZNF81	zinc finger protein 81	ENSG00000197779	0.58
HNRNPRP1	heterogeneous nuclear ribonucleoprotein R pseudogene 1	ENSG00000223984	0.58
CIDECP	cell death-inducing DFFA-like effector c pseudogene	ENSG00000186162	0.58
PRRC2C	proline rich coiled-coil 2C	ENSG00000117523	0.58
B9D2	B9 protein domain 2	ENSG00000123810	0.58
DPY19L3	dpy-19 like 3 (C. elegans)	ENSG00000178904	0.58
RP11-678G14.5		ENSG00000271182	0.58
MGAT4A	mannosyl (alpha-1,3-)-glycoprotein beta-1,4-N-acetylglucosaminyltransferase, isozyme A	ENSG00000071073	0.58
AC074117.10		ENSG00000234072	0.57
RAB11FIP1	RAB11 family interacting protein 1	ENSG00000156675	0.57
AGBL3	ATP/GTP binding protein like 3	ENSG00000146856	0.57
AIDA	axin interactor, dorsalization associated	ENSG00000186063	0.57
HEBP1	heme binding protein 1	ENSG00000013583	0.57
ISPD	isoprenoid synthase domain containing	ENSG00000214960	0.57
HES4	hes family bHLH transcription factor 4	ENSG00000188290	0.57
MRGBP	MRG domain binding protein	ENSG00000101189	0.57
C12orf49	chromosome 12 open reading frame 49	ENSG00000111412	0.57
BTF3L4P2	basic transcription factor 3 like 4 pseudogene 2	ENSG00000213189	0.56
LINC00176	long intergenic non-protein coding RNA 176	ENSG00000196421	0.56
SSH1	slingshot protein phosphatase 1	ENSG00000084112	0.56
IFIT5	interferon induced protein with tetratricopeptide repeats 5	ENSG00000152778	0.56
BLZF1	basic leucine zipper nuclear factor 1	ENSG00000117475	0.56
RBSN	rabenosyn, RAB effector	ENSG00000131381	0.56
LAP3P2	leucine aminopeptidase 3 pseudogene 2	ENSG00000213500	0.56
SYPL1	synaptophysin like 1	ENSG00000008282	0.56
KIF14	kinesin family member 14	ENSG00000118193	0.56
GADD45B	growth arrest and DNA damage inducible beta	ENSG00000099860	0.55
RP11-713C5.1		ENSG00000265579	0.55

SOBP	sine oculis binding protein homolog	ENSG00000112320	0.55
TRIQK	triple QxxK/R motif containing	ENSG00000205133	0.55
TMEM248	transmembrane protein 248	ENSG00000106609	0.55
EXOC2	exocyst complex component 2	ENSG00000112685	0.55
SOX9	SRY-box 9	ENSG00000125398	0.55
PRELID3A	PRELI domain containing 3A	ENSG00000141391	0.55
DDHD1	DDHD domain containing 1	ENSG00000100523	0.55
UBE2O	ubiquitin conjugating enzyme E2 O	ENSG00000175931	0.55
ATG9B	autophagy related 9B	ENSG00000181652	0.55
CCDC150	coiled-coil domain containing 150	ENSG00000144395	0.54
GAL	galanin and GMAP prepropeptide	ENSG00000069482	0.54
UTP4	UTP4, small subunit processome component	ENSG00000141076	0.54
TTC27	tetratricopeptide repeat domain 27	ENSG00000018699	0.54
KLHL11	kelch like family member 11	ENSG00000178502	0.54
SLC36A1	solute carrier family 36 member 1	ENSG00000123643	0.54
ADAM17	ADAM metallopeptidase domain 17	ENSG00000151694	0.54
ZNF326	zinc finger protein 326	ENSG00000162664	0.54
RDH11	retinol dehydrogenase 11 (all-trans/9-cis/11-cis)	ENSG00000072042	0.54
EIF5A2	eukaryotic translation initiation factor 5A2	ENSG00000163577	0.54
USP42	ubiquitin specific peptidase 42	ENSG00000106346	0.54
TMEM231	transmembrane protein 231	ENSG00000205084	0.54
RPGR	retinitis pigmentosa GTPase regulator	ENSG00000156313	0.53
HNRNPR	heterogeneous nuclear ribonucleoprotein R	ENSG00000282958	0.53
ZNF391	zinc finger protein 391	ENSG00000124613	0.53
RASIP1	Ras interacting protein 1	ENSG00000105538	0.53
TNPO1P3	transportin 1 pseudogene 3	ENSG00000229586	0.53
DTNB	dystrobrevin beta	ENSG00000138101	0.53
FAM89A	family with sequence similarity 89 member A	ENSG00000182118	0.53
BCOR	BCL6 corepressor	ENSG00000183337	0.53
RP11-295H24.3		ENSG00000259531	0.53
CARNS1	carnosine synthase 1	ENSG00000172508	0.53
THAP6	THAP domain containing 6	ENSG00000174796	0.53
PRKD2	protein kinase D2	ENSG00000105287	0.52
PKN3	protein kinase N3	ENSG00000160447	0.52
RP11-342K6.1		ENSG00000270696	0.52
DZIP1	DAZ interacting zinc finger protein 1	ENSG00000134874	0.52
PLEKHN1	pleckstrin homology domain containing N1	ENSG00000187583	0.52
BICDL2	BICD family like cargo adaptor 2	ENSG00000162069	0.52
LRRC24	leucine rich repeat containing 24	ENSG00000254402	0.52
PEX11A	peroxisomal biogenesis factor 11 alpha	ENSG00000166821	0.52
ZMAT3	zinc finger matrin-type 3	ENSG00000172667	0.52
AFAP1	actin filament associated protein 1	ENSG00000196526	0.52
DGCR11	DiGeorge syndrome critical region gene 11 (non-protein coding)	ENSG00000273311	0.52
BCAP29	B-cell receptor associated protein 29	ENSG00000075790	0.52

NUP35	nucleoporin 35	ENSG00000163002	0.51
RAB3B	RAB3B, member RAS oncogene family	ENSG00000169213	0.51
CCDC122	coiled-coil domain containing 122	ENSG00000151773	0.51
OSTM1	osteopetrosis associated transmembrane protein 1	ENSG00000081087	0.51
CLIC3	chloride intracellular channel 3	ENSG00000169583	0.51
IER5L	immediate early response 5 like	ENSG00000188483	0.51
CCDC136	coiled-coil domain containing 136	ENSG00000128596	0.51
MAP1A	microtubule associated protein 1A	ENSG00000166963	0.51
PHLDB3	pleckstrin homology like domain family B member 3	ENSG00000176531	0.51
ISCU	iron-sulfur cluster assembly enzyme	ENSG00000136003	0.51
GPM6B	glycoprotein M6B	ENSG00000046653	0.51
IL10RB	interleukin 10 receptor subunit beta	ENSG00000243646	0.51
SGPP1	sphingosine-1-phosphate phosphatase 1	ENSG00000126821	0.51
STAM2	signal transducing adaptor molecule 2	ENSG00000115145	0.51
SCML1	sex comb on midleg-like 1 (Drosophila)	ENSG00000047634	0.51
BMP8B	bone morphogenetic protein 8b	ENSG00000116985	0.51
TWIST1	twist family bHLH transcription factor 1	ENSG00000122691	0.51
HSPB1P2	heat shock protein family B (small) member 1 pseudogene 2	ENSG00000230216	0.51
WFS1	wolframin ER transmembrane glycoprotein	ENSG00000109501	0.50

Supplementary Table S3.

Gene	Ensemble Gene ID	Log_2FC	# miR-642a-5p sites
DOHH^^	ENSG00000129932	-0.69	6
TP53INP2	ENSG00000078804	-0.56	5
AAK1	ENSG00000115977	-0.78	4
CMTM4	ENSG00000183723	-0.71	4
MEX3A	ENSG00000254726	-0.51	4
TSPAN15	ENSG00000099282	-0.70	3
DR1	ENSG00000117505	-0.68	3
SLC7A2	ENSG00000003989	-0.56	3
WT1**	**ENSG00000184937**	**-0.55**	**3**
ZDHHC5	ENSG00000156599	-0.52	3
RBMS2	ENSG00000076067	-0.77	2
MEF2D	ENSG00000116604	-0.76	2
NUAK1**	**ENSG00000074590**	**-0.76**	**2**
IGSF3	ENSG00000143061	-0.75	2
PCNP	ENSG00000081154	-0.65	2
PCYOX1L	ENSG00000145882	-0.63	2
FAM134C	ENSG00000141699	-0.62	2
PRKAA2	ENSG00000162409	-0.62	2
NCBP2	ENSG00000114503	-0.61	2
NUDT21	ENSG00000167005	-0.60	2
RHOBTB3	ENSG00000164292	-0.58	2
PLEKHM3	ENSG00000178385	-0.58	2
FXN	ENSG00000165060	-0.57	2
APMAP	ENSG00000101474	-0.56	2
ABHD10	ENSG00000144827	-0.55	2
CD2AP	ENSG00000198087	-0.54	2
PGGT1B	ENSG00000164219	-0.54	2
SIRT5	ENSG00000124523	-0.53	2
NHLRC3	ENSG00000188811	-0.51	2
DQX1	ENSG00000144045	-1.08	1
LAMP1	ENSG00000185896	-0.92	1
USP12	ENSG00000152484	-0.87	1
DIRC2	ENSG00000138463	-0.85	1
PROCA1	ENSG00000167525	-0.84	1
NQO1	ENSG00000181019	-0.83	1
IRF1	ENSG00000125347	-0.79	1
TLE6	ENSG00000104953	-0.77	1
RAB6A	ENSG00000175582	-0.74	1
C4orf3	ENSG00000164096	-0.71	1
SPINT2	ENSG00000167642	-0.70	1
APLF	ENSG00000169621	-0.70	1

STRAP	ENSG00000023734	-0.69	1
SLC16A6	ENSG00000108932	-0.69	1
NECAP1	ENSG00000089818	-0.66	1
MANBA	ENSG00000109323	-0.64	1
CERS2	ENSG00000143418	-0.64	1
TTC7B	ENSG00000165914	-0.63	1
SUV39H1	ENSG00000101945	-0.62	1
ZFAND6	ENSG00000086666	-0.62	1
NCK2	ENSG00000071051	-0.62	1
CASC4	ENSG00000166734	-0.61	1
MFHAS1	ENSG00000147324	-0.61	1
RMDN3	ENSG00000137824	-0.60	1
TMEM183A	ENSG00000163444	-0.60	1
ACBD7	ENSG00000176244	-0.60	1
KHDC1	ENSG00000135314	-0.58	1
ZNF354C	ENSG00000177932	-0.58	1
LARS2	ENSG00000011376	-0.57	1
KLHL23	ENSG00000213160	-0.57	1
FAM120C	ENSG00000184083	-0.56	1
RASSF3**	**ENSG00000153179**	**-0.54**	**1**
FOXN2	ENSG00000170802	-0.53	1
PPP3CA	ENSG00000138814	-0.53	1
IGHMBP2	ENSG00000132740	-0.53	1
KLF12	ENSG00000118922	-0.52	1
CDC37L1	ENSG00000106993	-0.52	1
ANAPC13	ENSG00000129055	-0.52	1
BZW1	ENSG00000082153	-0.51	1
DCAF16	ENSG00000163257	-0.51	1
SKP2**	**ENSG00000145604**	**-0.50**	**1**
TMEM123	ENSG00000152558	-0.50	1
POLR2M	ENSG00000255529	-0.50	1

Supplementary Table S4.

Gene	Ensemble Gene ID	Log_2 FC	# miR-642a-5p sites
RAB11FIP1	ENSG00000156675	0.57	3
C12orf49	ENSG00000111412	0.57	3
PRKD2	ENSG00000105287	0.52	3
ZNF788	ENSG00000214189	0.88	2
DDN	ENSG00000181418	0.74	2
MMP17	ENSG00000198598	0.72	2
STAT3	ENSG00000168610	0.65	2
AGBL3	ENSG00000146856	0.57	2
SSH1	ENSG00000084112	0.56	2
ZNF326	ENSG00000162664	0.54	2
GIPC3	ENSG00000179855	1.10	1
CAMKV	ENSG00000164076	1.02	1
MFAP3L	ENSG00000198948	0.88	1
TMEM158	ENSG00000249992	0.86	1
CELSR2	ENSG00000143126	0.80	1
SPATA6	ENSG00000132122	0.79	1
SPOPL	ENSG00000144228	0.77	1
EMC10	ENSG00000161671	0.74	1
IZUMO4	ENSG00000099840	0.74	1
UBTD1	ENSG00000165886	0.71	1
DTX3L	ENSG00000163840	0.70	1
KLRG2	ENSG00000188883	0.67	1
NARF	ENSG00000141562	0.66	1
PROSC	ENSG00000147471	0.66	1
LLPH	ENSG00000139233	0.65	1
ZXDA	ENSG00000198205	0.64	1
ZNF225	ENSG00000256294	0.63	1
SLC45A4	ENSG00000022567	0.63	1
SEMA3G	ENSG00000010319	0.63	1
TERT	ENSG00000164362	0.61	1
ZFPM1	ENSG00000179588	0.61	1
ALPK1	ENSG00000073331	0.60	1
CTU1	ENSG00000142544	0.59	1
RPS6KA2	ENSG00000071242	0.59	1
ZNF81	ENSG00000197779	0.58	1
SOBP	ENSG00000112320	0.55	1
DDHD1	ENSG00000100523	0.55	1
ATG9B	ENSG00000181652	0.55	1
SLC36A1	ENSG00000123643	0.54	1
TMEM231	ENSG00000205084	0.54	1
ZNF391	ENSG00000124613	0.53	1

DZIP1	ENSG00000134874	0.52	1
ZMAT3	ENSG00000172667	0.52	1
RAB3B	ENSG00000169213	0.51	1
IER5L	ENSG00000188483	0.51	1
MAP1A	ENSG00000166963	0.51	1
TWIST1	ENSG00000122691	0.51	1
